# Supplementary material for: Summer warming explains widespread but not uniform greening in the Arctic tundra biome
Source: Nat Commun. 2020 Sep 22;11:4621. doi: 10.1038/s41467-020-18479-5 (PMC7509805; doi:10.1038/s41467-020-18479-5)
Supplement: Supplementary file 1 — Supplementary Information [file 41467_2020_18479_MOESM1_ESM.pdf]

Supplementary Information:
Summer warming explains widespread but not uniform greening in the Arctic tundra biome

Berner et al.

Supplementary Figures

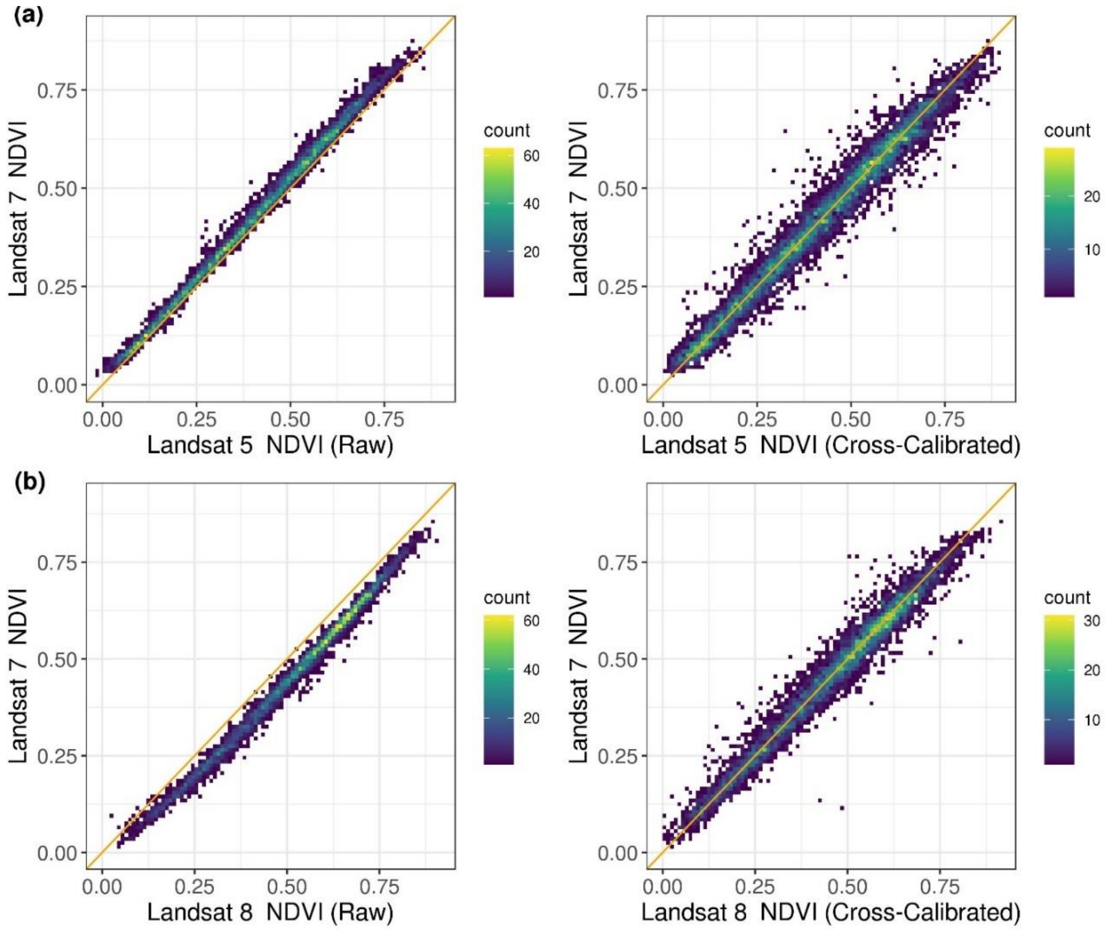

Supplementary Figure 1 | Differences in NDVI among Landsat sensor before and after cross-sensor calibration. Landsat 7 NDVI compared with raw and cross-calibrated (a) Landsat 5 NDVI and (b) Landsat 8 NDVI. Note that raw Landsat 5 NDVI was consistently lower than Landsat 7 NDVI, which was consistently lower than Landsat 8 NDVI (left columns). This can introduce an artificial positive trend in composite NDVI time series. This issue was obviated by further cross-sensor calibration using Random Forest machine learning algorithms (right columns). Each data point is an estimate of 15-day median NDVI computed from observations acquired during the years of overlap between pairs of sensors at each sampling site. Each sampling site contributes a single data point with the 15-day period selected at random from available periods during summers with at least five observations. The diagonal orange lines show 1:1 relationships.

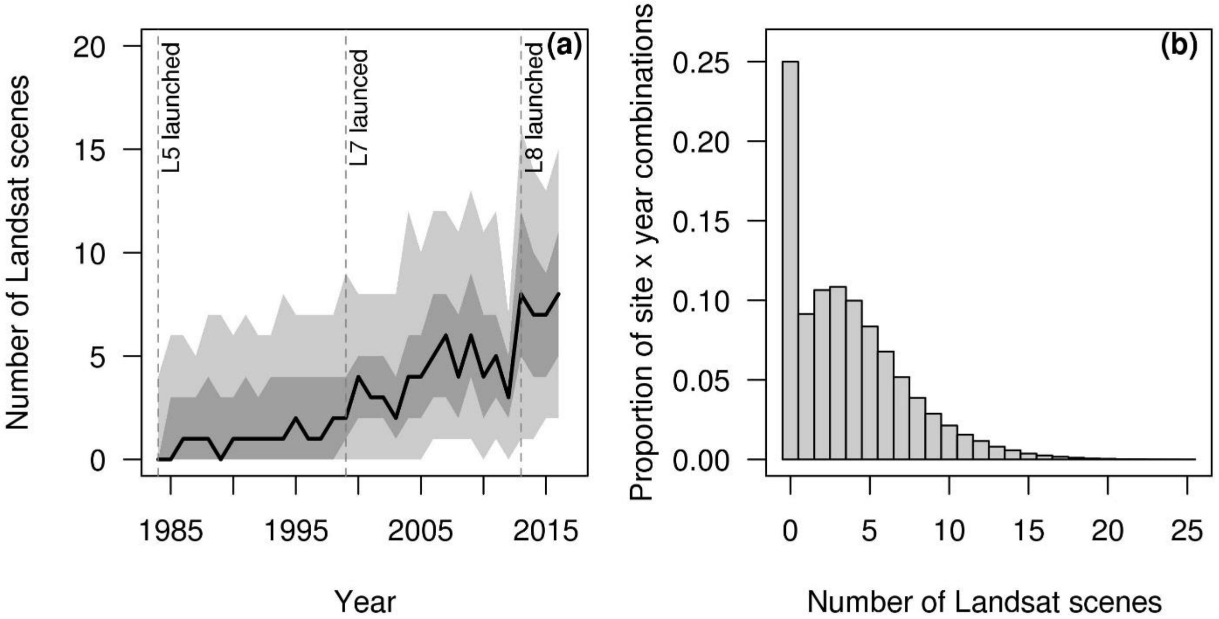

Supplementary Figure 2 | Summary of Landsat data availability from 1984 to 2016 assessed using a random sampling sites in the Arctic. (a) Median number [black line] of cloud- and snow-free Landsat scenes acquired each summer (June through August). The availability of useable Landsat scenes increased though time with the launch of successive satellites. Shaded bands encompass 50% [dark gray] and 90% [dark gray] of sampling sites. (b) Histogram depicting availability of summer Landsat scene across all sampling sites and years.

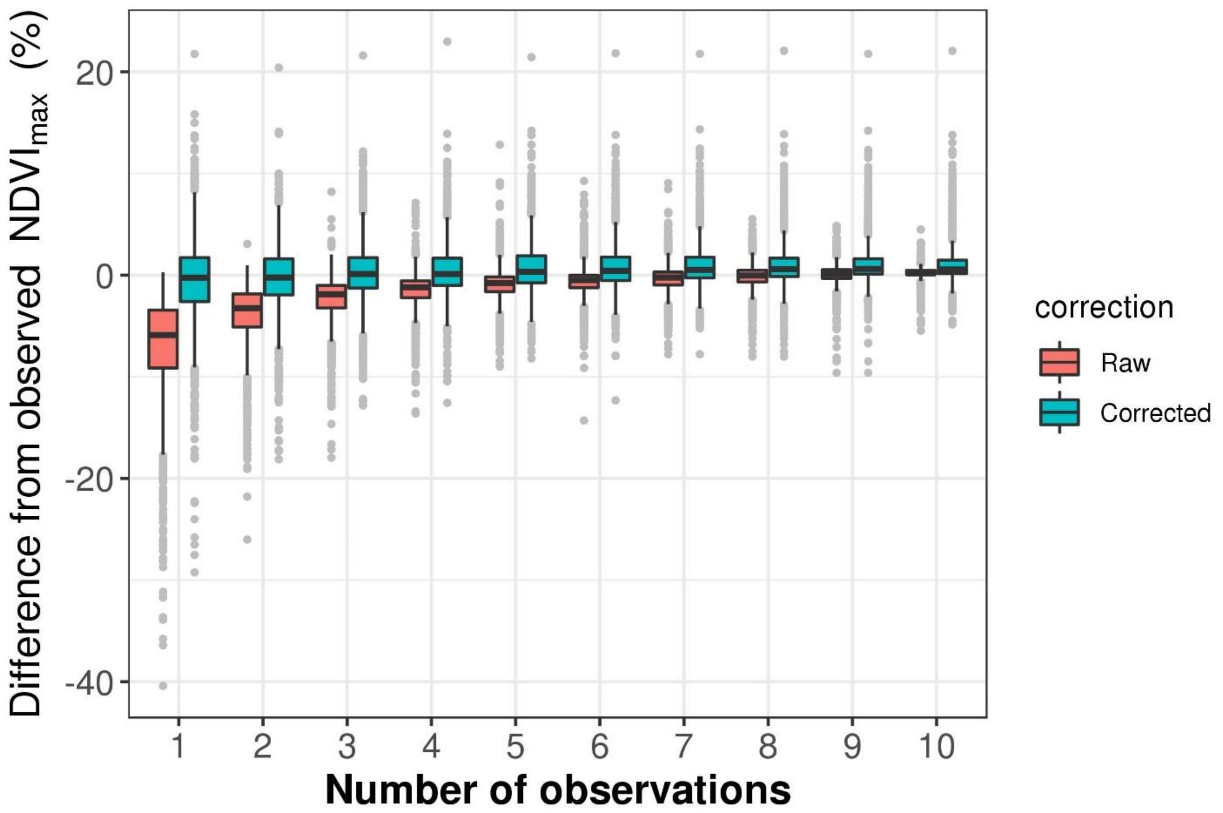

Supplementary Figure 3 | Assessment of how estimates of maximum summer NDVI ($NDVI_{max}$) are affected by the number of Landsat scenes available from a summer. Estimates of $NDVI_{max}$ increase asymptotically with scene availability when derived from raw (uncorrected) Landsat observations; however, estimates of $NDVI_{max}$ exhibit minimal change with scene availability when Landsat observations are corrected using site-specific information on land surface phenology. Intra-box lines denote median percent error among *site x years* that went into the analysis, while boxes encompass 50% of observations, and whiskers extend 1.5 times the interquartile range.

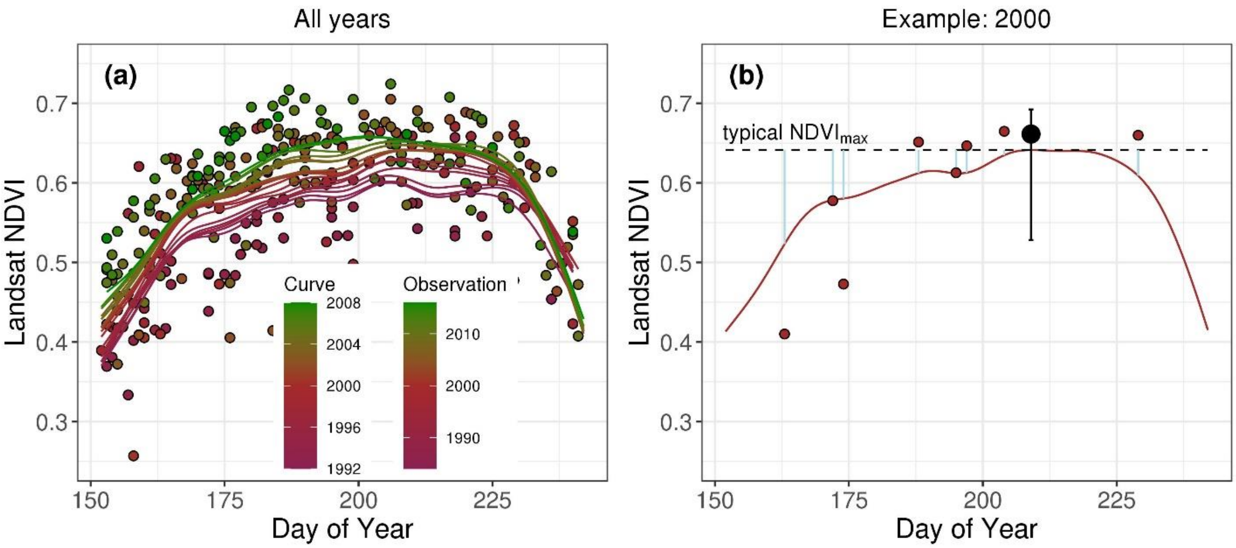

Supplementary Figure 4 | Illustration of approach for estimating annual Landsat maximum summer NDVI ($NDVI_{max}$). **(a)** Seasonal progression of Landsat NDVI from June through August for a sampling site in the Arctic. Each point is a quality-controlled Landsat 5, 7, or 8 observation from 1985 to 2016. Each curve depicts the typical land surface phenology for a 17-year period derived by fitting a cubic spline through all observations from that period. **(b)** Annual Landsat $NDVI_{max}$ (black point) was estimated using each summer observation (brown points) together with phenological information on the typical difference in NDVI between peak summer and the timing of each observation (blue lines). Specifically, the black point represents the median $NDVI_{max}$ estimated from all summer observation, while the error bar encompasses the full range of estimates.

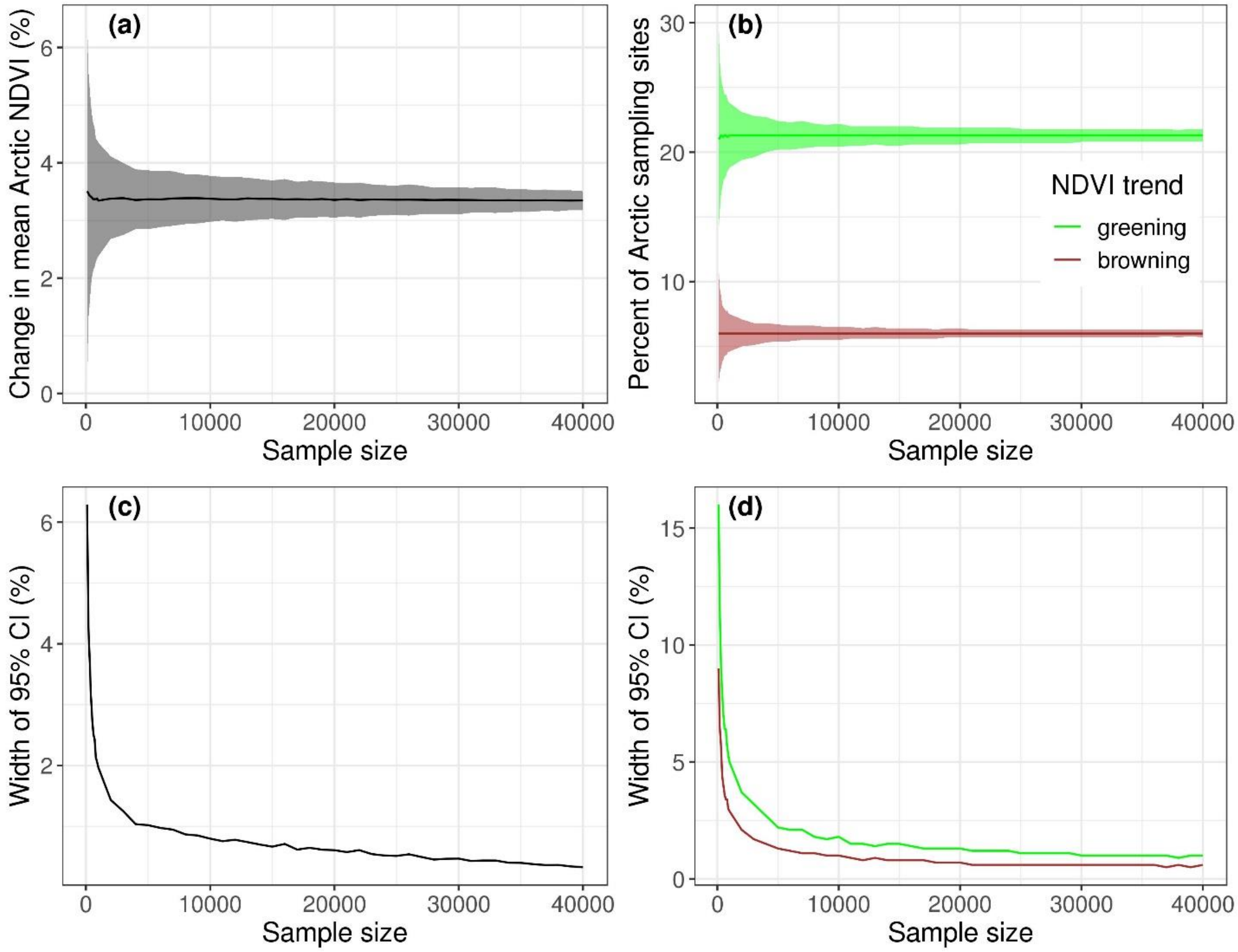

Supplementary Figure 5 | Effects of sample size on estimates of Landsat $NDVI_{max}$ trends in the Arctic from 2000 to 2016. Trend metrics include **(a)** the relative change in mean Arctic $NDVI_{max}$ (%) and **(b)** the percentage of sites with a positive ("greening") or negative ("browning") trend in $NDVI_{max}$ ($\alpha = 0.10$). Solid lines depict median estimates from 10^3 Monte Carlo simulations while error bands depict 95% confidence intervals (CI). Changes in the width of the 95% CIs are shown in panels **(c)** and **(d)**. Each simulation not only used random subsets of sites, but also $NDVI_{max}$ time series generated with randomly permuted surface reflectance, cross-sensor calibration models, phenological-correction parameters.

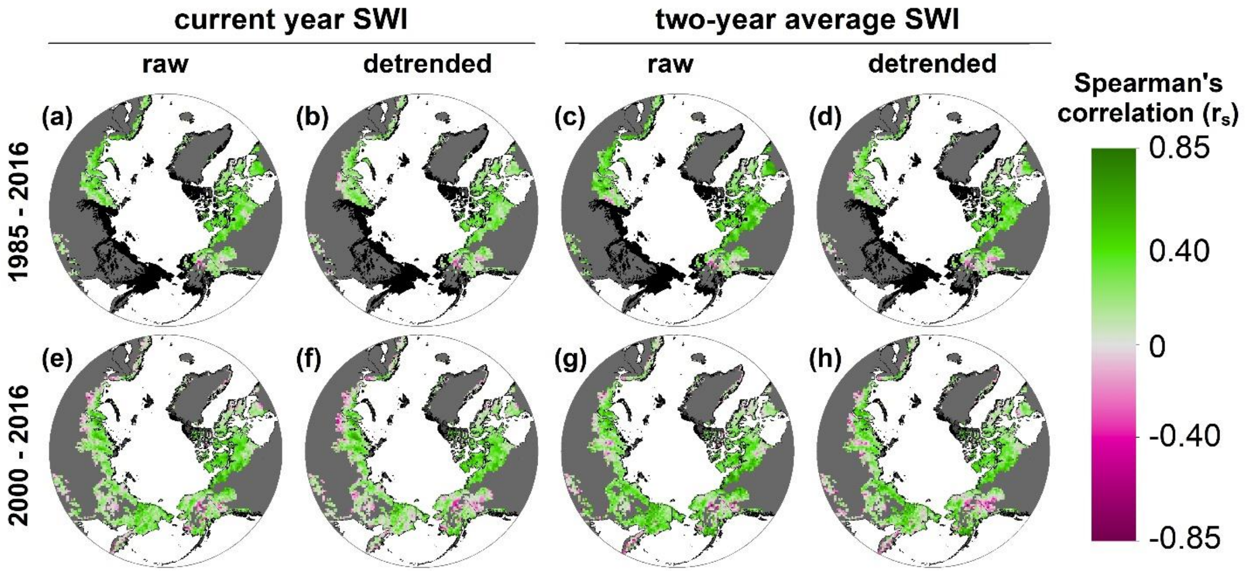

Supplementary Figure 6 | Correlations between annual Landsat NDVI$_{max}$ [unitless] and the summer warmth index [SWI; °C] across the Arctic during recent decades. Mean Spearman's correlation (r_s) between annual NDVI$_{max}$ and SWI among sites within each 50 x 50 km grid cell. Each grid cell shows the mean Spearman's correlation (r_s) between annual NDVI$_{max}$ and SWI among sites. Specifically, annual NDVI$_{max}$ was correlated with either current-year and two-year average SWI, and then reassessed after linearly detrending both NDVI$_{max}$ and SWI time series. Note that annual NDVI$_{max}$ was derived by averaging the annual time series from sites within each grid cell (50 x 50 km resolution). Each grid cell depicts the median correlation coefficient derived from 10^3 Monte Carlo simulations that randomly permuted both NDVI$_{max}$ and SWI timeseries.

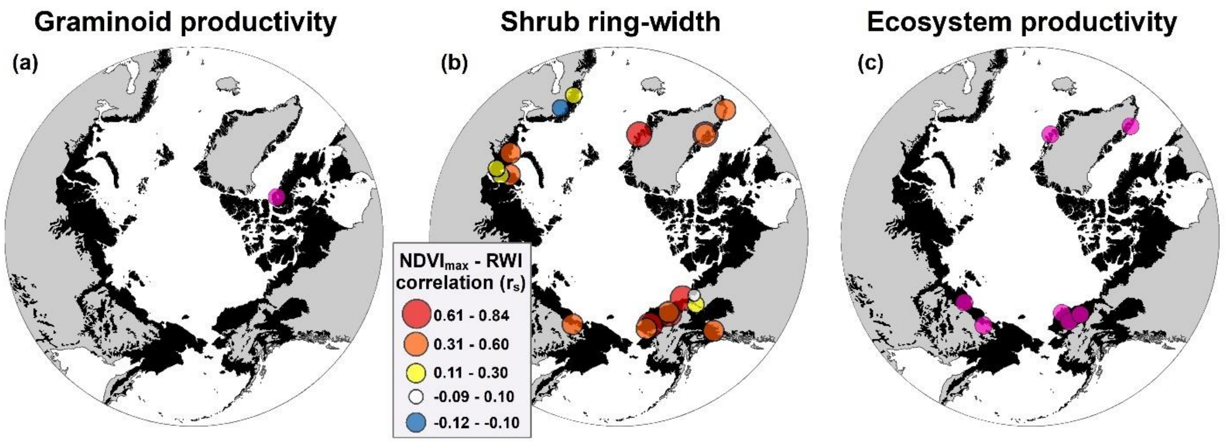

Supplementary Figure 7 | Location of field sites that provide metrics of plant productivity that were compared with Landsat NDVI$_{max}$. Field data sets include graminoid productivity **(a)**, shrub ring-width **(b)**, and ecosystem gross primary productivity estimated from measurements made by eddy covariance flux towers **(c)**. The size of each shrub ring-width plotting symbol is proportional to the Spearman correlation (r_s) between Landsat NDVI$_{max}$ and the ring-width index chronology at that location.

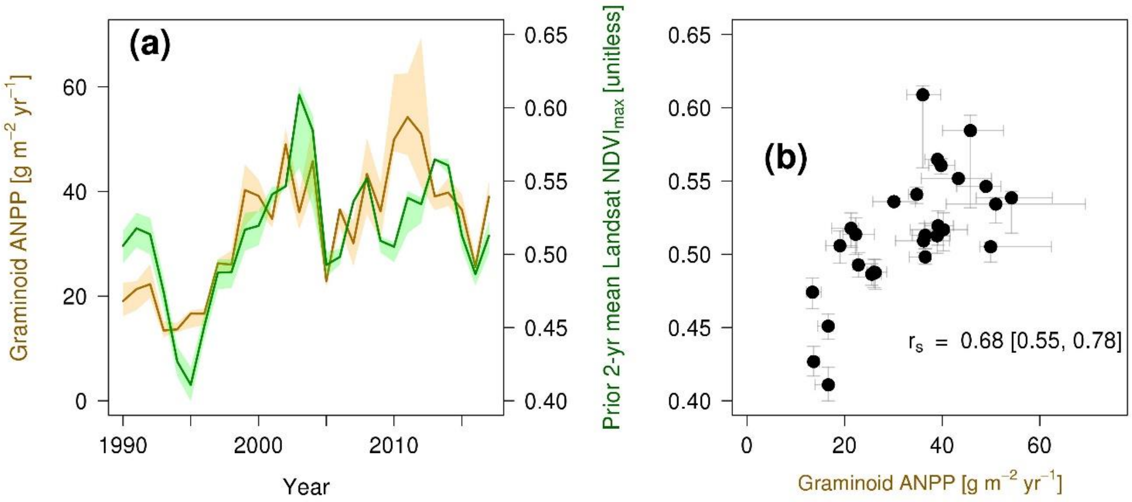

Supplementary Figure 8 | Graminoid productivity and Landsat $NDVI_{max}$ on Bylot Island. Time series **(a)** and scatter plot **(b)** of annual median graminoid aboveground net primary productivity [ANPP] and Landsat $NDVI_{max}$ [unitless] from 1990 to 2017 at long-term monitoring sites on Bylot Island in northern Canada. Lines **(a)** and points **(b)** depict medians and error bands **(a)** and bars **(b)** depict 95% confidence intervals derived from 10^3 Monte Carlo simulations. There were typically 12 quadrats harvested per year, but 11 quadrats in 1991, 2013, 2014, and 2016. Quadrats were harvested at four subsites over this period. Annual median Landsat $NDVI_{max}$ was computed using data from these four subsites.

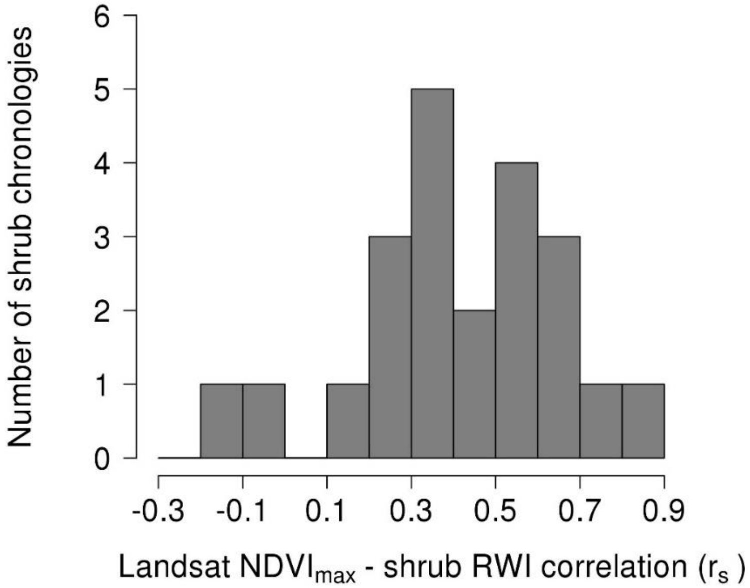

Supplementary Figure 9 | Summary of correlations between annual shrub growth and Landsat $NDVI_{max}$. Frequency distribution of Spearman correlations (r_s) between annual detrended Landsat $NDVI_{max}$ and shrub RWI chronologies. The correlation for each chronology represents the median r_s of 10^3 Monte Carlo simulations.

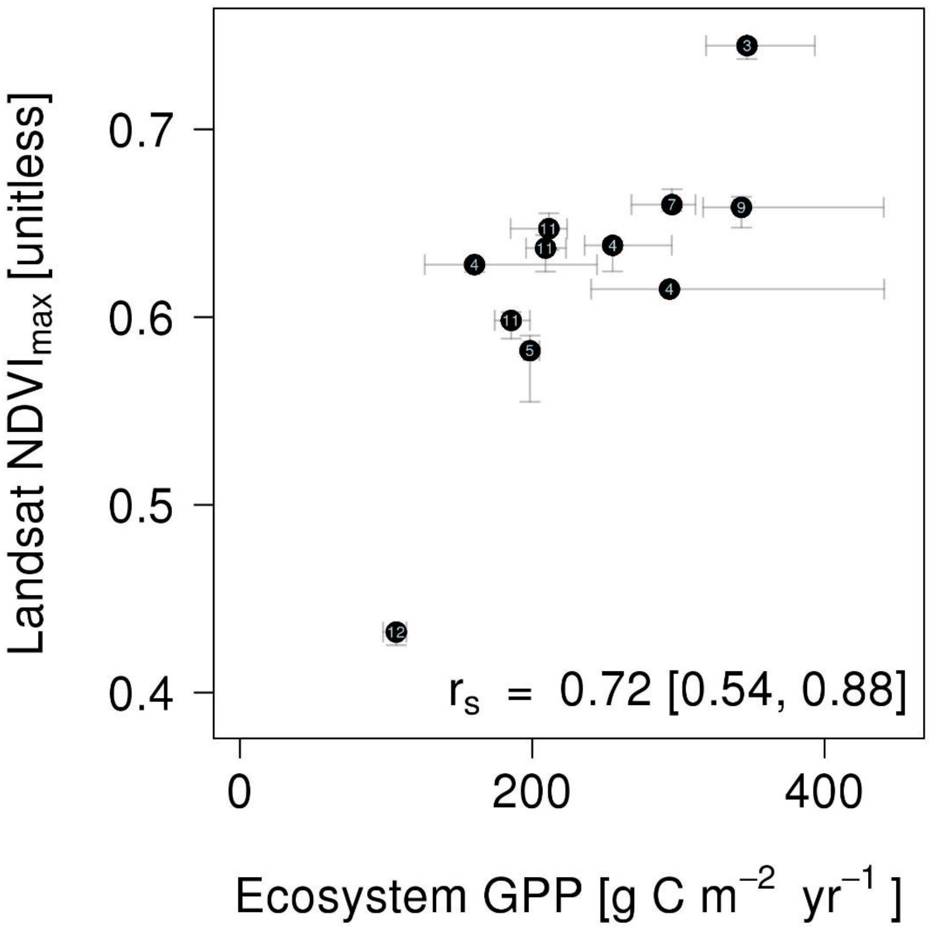

Supplementary Figure 10 | Relationship between median annual Landsat $NDVI_{max}$ and ecosystem gross primary productivity (GPP) across 11 flux towers in the Arctic. Each point depicts medians computed over the number of years specified in white text within each point. Error bars represent 95% confidence intervals (CI) derived from 10^3 Monte Carlo simulations. The Spearman correlation (r_s) also includes a 95% CI. Supplementary Table 12 provides additional details about each site.

Supplementary Tables

Sensor	Random Forest Out-of-Bag Evaluation			Cross-Validation			
	r^2	RMSE	N sites	r^2	RMSE	Bias	N sites
Landsat 5	0.969 [0.968, 0.970]	0.036 [0.035, 0.037]	16,599	0.969 [0.967, 0.971]	0.036 [0.035, 0.037]	0 [-0.001, 0.001]	8,176
Landsat 8	0.967 [0.965, 0.968]	0.033 [0.033, 0.034]	12,820	0.967 [0.965, 0.969]	0.033 [0.032, 0.034]	0 [-0.001, 0.001]	6,314

Supplementary Table 1 | Performance of Random Forest models used to cross-calibrate NDVI from Landsat 5 and 8 with Landsat 7. Model performance was nearly identical whether assessed using the Random Forest out-of-bag evaluation or external cross-validation. Evaluation criteria include the coefficient of variance (r^2), root mean squared error (RMSE), and bias. Each metric is accompanied by a 95% confidence interval derived from Monte Carlo simulations ($n = 10^3$).

Period	Domain	Landsat $NDVI_{max}$ trend		
		**Δ $NDVI_{max}$ (unitless)**	**Δ $NDVI_{max}$ (%)**	**tau**
1985-2016	Arctic	0.036 [0.034, 0.037]	7.3 [7.0, 7.7]	0.41 [0.40, 0.42]
	High Arctic	0.007 [0.004, 0.010]	2.1 [1.4, 2.9]	0.14 [0.11, 0.17]
	Low Arctic	0.034 [0.033, 0.036]	7.1 [6.7, 7.4]	0.36 [0.34, 0.38]
	Oro Arctic	0.037 [0.036, 0.039]	6.0 [5.7, 6.3]	0.51 [0.49, 0.54]
2000-2016	Arctic	0.019 [0.018, 0.020]	3.6 [3.4, 3.7]	0.53 [0.48, 0.57]
	High Arctic	0.024 [0.021, 0.028]	8.0 [6.7, 9.3]	0.42 [0.38, 0.45]
	Low Arctic	0.030 [0.029, 0.031]	6.0 [5.8, 6.1]	0.55 [0.52, 0.57]
	Oro Arctic	0.011 [0.010, 0.012]	1.8 [1.6, 1.9]	0.63 [0.58, 0.67]

Supplementary Table 2 | Changes in mean tundra greenness for the Arctic and each bioclimatic zone during recent decades. Trends in mean Landsat $NDVI_{max}$ (unitless) were assessed for the Arctic and each bioclimatic zone over two time periods (1985 to 2016 and 2000 to 2016) using Theil-Sen slope estimators and Mann-Kendall trend tests. Each trend includes the total absolute and relative change and a tau statistic. Each metric is accompanied by a 95% confidence interval derived from 10^3 Monte Carlo simulations.

Period	Domain	Number of sampling sites	Percent of sampling sites			Ratio of Greening to Browning
			Greening	Browning	No trend	
1985-2016	Arctic	21992 [21503, 22488]	37.3 [36.3, 38.4]	4.7 [4.4, 5.1]	58.0 [57.1, 58.7]	7.9 [7.1, 8.7] : 1
	High Arctic	4801 [4594, 5010]	24.9 [23.1, 26.7]	6.2 [5.3, 7.2]	68.9 [67.4, 70.4]	4.0 [3.3, 4.9] : 1
	Low Arctic	10894 [10603, 11185]	41.6 [40.4, 42.8]	3.8 [3.4, 4.3]	54.6 [53.6, 55.6]	10.9 [9.6, 12.5] : 1
	Oro Arctic	6295 [6109, 6489]	39.4 [38.1, 40.8]	5.1 [4.7, 5.6]	55.5 [54.3, 56.6]	7.7 [6.9, 8.6] : 1
2000-2016	Arctic	41886 [41402, 42357]	21.3 [20.8, 21.7]	6.0 [5.8, 6.3]	72.7 [72.3, 73.1]	3.6 [3.4, 3.8] : 1
	High Arctic	6288 [6119, 6476]	11.4 [10.5, 12.5]	7.4 [6.7, 8.3]	81.1 [80.2, 82.1]	1.5 [1.3, 1.8] : 1
	Low Arctic	18824 [18543, 19098]	21.3 [20.7, 21.8]	4.9 [4.6, 5.2]	73.8 [73.2, 74.4]	4.3 [4.0, 4.6] : 1
	Oro Arctic	16771 [16507, 17038]	25.0 [24.4, 25.7]	6.6 [6.3, 7.0]	68.4 [67.7, 68.9]	3.8 [3.5, 4.0] : 1

Supplementary Table 3 | Frequency of recent changes in tundra greenness among sampling sites in the Arctic and each bioclimatic zone. For each sampling sites, the Landsat $NDVI_{max}$ (unitless) trend was classified as greening, browning, or no trend based on the significance (α = 0.10) of Mann-Kendall trend tests and direction of Theil-Sen slope from 1985 to 2016 and 2000 to 2016. The percentage of sites with greening, browning, or no trend are summarized for the Arctic and each bioclimatic zone. Each metric is accompanied by a 95% confidence interval derived from 10^3 Monte Carlo simulations.

Number of sample sites	Change in mean Arctic NDVI (%)	Percent of sampling sites		
		Browning	Greening	No trend
10^2	3.51 [0.14, 6.43]	6.00 [2.00, 11.00]	21.00 [14.00, 30.00]	73.00 [63.00, 81.00]
10^3	3.34 [2.38, 4.35]	6.00 [4.60, 7.50]	21.30 [18.80, 23.80]	72.70 [70.00, 75.40]
10^4	3.38 [2.98, 3.77]	6.00 [5.50, 6.50]	21.30 [20.40, 22.20]	72.70 [71.90, 73.60]
$4x10^4$	3.35 [3.18, 3.51]	6.00 [5.70, 6.30]	21.30 [20.80, 21.80]	72.70 [72.30, 73.10]

Supplementary Table 4 | Effects of sample size on estimates of Landsat $NDVI_{max}$ trends in the Arctic from 2000 to 2016. Trend characteristics include the total relative change in mean Arctic $NDVI_{max}$ and the percentage of sites with a positive ("greening"), negative ("browning"), or no trend in $NDVI_{max}$ ($\alpha = 0.10$). Each trend metric represents the median estimate from 10^3 Monte Carlo simulations and is accompanied by a 95% confidence interval (CI). Note that each median trend metric is quite stable across a 400-fold range in sample size and that the width of each 95% CI asymptotically decreases.

Data Set	Version	Resolution	Period	Ref.
Berkeley Earth	2018	1.0 x 1.0 °	1850 – 2018	[1]
Univ. York HadCRUT4 with UAH	2.0	5.0 x 5.0 °	1979 – 2017	[2]
NASA GISS Surface Temp. Analysis	2018	2.0 x 2.0 °	1880 – 2018	[3, 4]
Univ. East Anglia Climate Research Unit	4.01	0.5 x 0.5 °	1901 – 2016	[5]
Univ. Delaware	5.01	0.5 x 0.5 °	1900 – 2017	[6]

Supplementary Table 5 | Summary of air temperature data sets used in the analysis.

**Period**	**Domain**	**SWI trend**	
		**Δ °C**	**tau**
1985-2016	Arctic	5.0 [4.9, 5.1]	0.61 [0.60, 0.63]
	High Arctic	3.6 [3.3, 3.9]	0.46 [0.43, 0.50]
	Low Arctic	5.1 [5.0, 5.3]	0.60 [0.58, 0.61]
	Oro Arctic	5.6 [5.4, 5.8]	0.59 [0.56, 0.60]
2000-2016	Arctic	2.5 [2.3, 2.7]	0.37 [0.33, 0.40]
	High Arctic	2.6 [2.1, 3.1]	0.27 [0.18, 0.35]
	Low Arctic	2.2 [1.9, 2.5]	0.30 [0.25, 0.33]
	Oro Arctic	2.6 [2.3, 3.0]	0.40 [0.35, 0.47]

Supplementary Table 6 | Changes in summer air temperatures for the Arctic and each bioclimatic zone during recent decades. Trends in the mean summer warmth index (SWI; °C) were assessed for the Arctic and each bioclimatic zone over two time periods (1985 to 2016 and 2000 to 2016) using Theil-Sen slope estimators and Mann-Kendall trend tests. Each trend includes the total change and a tau statistic accompanied by 95% confidence intervals derived from 10^3 Monte Carlo simulations based on an ensemble of five temperature data sets.

Period	Domain	Spearman correlation (r_s) between $NDVI_{max}$ and …			
		current year SWI		two-year average SWI	
		with trends	detrended	with trends	detrended
1985-2016	Arctic	0.68 [0.66,0.70]	0.43 [0.41,0.45]	0.86 [0.85,0.88]	0.72 [0.69,0.75]
	High Arctic	0.63 [0.58,0.67]	0.58 [0.53,0.62]	0.73 [0.69,0.77]	0.65 [0.58,0.72]
	Low Arctic	0.61 [0.58,0.63]	0.31 [0.27,0.34]	0.83 [0.81,0.84]	0.63 [0.61,0.65]
	Oro Arctic	0.69 [0.66,0.71]	0.40 [0.36,0.45]	0.77 [0.75,0.79]	0.43 [0.40,0.47]
2000-2016	Arctic	0.76 [0.73,0.78]	0.39 [0.33,0.46]	0.89 [0.88,0.91]	0.68 [0.65,0.70]
	High Arctic	0.75 [0.69,0.80]	0.78 [0.72,0.85]	0.70 [0.64,0.77]	0.70 [0.61,0.77]
	Low Arctic	0.65 [0.61,0.69]	0.38 [0.33,0.44]	0.84 [0.78,0.88]	0.51 [0.46,0.56]
	Oro Arctic	0.70 [0.68,0.72]	0.46 [0.40,0.51]	0.88 [0.85,0.92]	0.52 [0.50,0.57]

Supplementary Table 7 | Correlations between annual mean tundra greenness (Landsat $NDVI_{max}$; unitless) and summer air temperatures (SWI; °C) for the Arctic and each bioclimatic zone during recent decades. Spearman correlations (r_s) were used to assess co-variation between mean $NDVI_{max}$ and both current year and 2-year average SWI. Co-variation was also assessed after linearly detrending both $NDVI_{max}$ and SWI time series. Each correlation coefficient is accompanied by a 95% confidence interval derived from 10^3 Monte Carlo simulations.

Theme	Variable	Units	Period	Cadence	Resolution	Ref.
Climate	Summer warmth index	°C	2000-2016	Annual	50 km	Derived from [1, 2, 4, 5, 6]
	Min. summer soil moisture	mm	2000-2016	Annual	4 km	[7]
Permafrost	Active layer thickness	cm	2003-2016	Annual	1 km	[8]
	Soil temperature (1 m)	°C	2003-2016	Annual	1 km	[9]
	Permafrost extent	%	2003-2016	Annual	1 km	[10]
	Thermokarst vulnerability	category	ca. 2015	Single time	--	[11]
Fire	Burned area	category	2001-2016	Annual	0.5 km	[12]
Topography	Elevation	m	ca. 2015	Single time	0.09 km	[13]
	Slope	°	ca. 2015	Single time	0.09 km	Derived from [13]
	Aspect	°	ca. 2015	Single time	0.09 km	Derived from [13]
	Topographic roughness	unitless	ca. 2015	Single time	0.09 km	Derived from [13]
	Topographic position	unitless	ca. 2015	Single time	0.09 km	Derived from [13]
Biological	Land cover	category	2015	Single time	0.30 km	[14]

Supplementary Table 8 | Summary of environmental data sets used with random forest models to predict Landsat $NDVI_{max}$ trends from 2000 to 2016 at each sampling site. These geospatial data sets span the pan-Arctic domain.

**$NDVI_{max}$ trend**	**Sensitivity**	**Specificity**	**Balanced Accuracy**
Browning	65.6 [61.1, 69.9] %	79.6 [76.5, 82.3] %	72.6 [70.3, 74.5] %
No trend	38.4 [33.4, 43.2] %	75.5 [71.7, 79.0] %	56.9 [54.6, 59.0] %
Greening	62.3 [57.1, 66.9] %	78.1 [75.2, 80.9] %	70.2 [67.9, 72.5] %

Supplementary Table 9 | Class-specific performance of Random Forest models used to predict the Landsat $NDVI_{max}$ trend from 2000 to 2016 at each sampling site. The Landsat $NDVI_{max}$ trend at each sampling site was classified as browning, no trend, or greening based on the direction and significance ($\alpha = 0.10$) of trend evaluated using a Mann-Kendal Trend Test and Theil-Sen Slope. A Random Forest model was fit for each of the 10^3 Monte Carlo simulations after balancing the number of sampling sites in each trend class. The performance of each model was assessed by withholding a random 33.3% of data for cross-validation. The overall cross-validated model classification accuracy was 55.4 [53.1, 57.5] %. The table summarizes the median and 95% confidence intervals for class-specific model sensitivity, specificity, and balanced accuracy derived from these simulations.

**Observed $NDVI_{max}$ trend**	**Predicted $NDVI_{max}$ trend**		
	Browning	No trend	Greening
Browning	431 [394, 465]	153 [121, 183]	74 [55, 92]
No trend	190 [160, 225]	252 [222, 281]	214 [178, 250]
Greening	77 [60, 100]	170 [135, 207]	410 [371, 449]

Supplementary Table 10 | Confusion matrix comparing observed and predicted Landsat $NDVI_{max}$ trends at sampling sites with predictions derived from Random Forest models. The Landsat $NDVI_{max}$ trend at each sampling site was classified as browning, no trend, or greening based on the direction and significance ($\alpha = 0.10$) of trend evaluated using Mann-Kendal Trend Test and Theil-Sen Slope. A confusion matrix was generated for each random forest model (10^3 Monte Carlo simulations) by withholding a random 33.3% of data for cross-validation. The table summarizes the median [95% CI] number of sampling sites falling in each category.

Country	Study area	Genus	Lat.	Lon.	rbar	r_s	n yrs.	n shrubs	Ref.
Canada	Dempster	Salix	67.045	-136.185	0.19 [0.17,0.22]	0.31 [0.05,0.55]	23	33 (19-41)	15
	Herschel Island	Salix	69.57	-138.901	0.08 [0.05,0.10]	0.61 [0.41,0.75]	21	48 (20-67)	15
	Kluane	Salix	61.214	-138.164	0.26 [0.24,0.27]	0.38 [0.23,0.53]	25	200 (56-297)	15
	Nowell Lake	Alnus	68.536	-133.646	0.30 [0.27,0.32]	-0.08 [-0.27,0.18]	21	32 (6-40)	15
Finland	Enontekio	Salix	68.628	24.79	0.54 [0.51,0.57]	-0.12 [-0.33,0.04]	28	17 (16-17)	16, 17
	Arsuk Fjord	Alnus	61.314	-48.109	0.47 [0.44,0.48]	0.30 [0.06,0.61]	16	25 (22-26)	15
Greenland	Kangerlussuaq	Betula	67.113	-50.326	0.28 [0.25,0.29]	0.61 [0.45,0.74]	18	30 (9-42)	18
	Kangerlussuaq	Salix	67.113	-50.326	0.48 [0.47,0.50]	0.60 [0.39,0.78]	18	26 (9-32)	19
	Zackenberg	Salix	74.466	-20.592	0.12 [0.10,0.14]	0.61 [0.48,0.74]	23	62 (18-87)	15
Russia	Bovanenkovo	Salix	70.394	68.436	0.67 [0.66,0.68]	0.33 [0.19,0.60]	24	27 (23-28)	16, 17
	Cherskii	Alnus	68.742	161.414	0.56 [0.55,0.57]	0.60 [0.49,0.73]	18	58 (55-60)	16, 17
	Laboravaya	Alnus	67.692	67.968	0.63 [0.62,0.65]	0.24 [0.03,0.44]	22	21 (20-24)	16, 17
	Laboravaya	Salix	67.692	67.968	0.57 [0.55,0.58]	0.25 [0.03,0.48]	22	28 (26-29)	16, 17
	Mordy Yaha	Salix	70.194	68.568	0.68 [0.66,0.68]	0.46 [0.30,0.59]	24	31 (29-33)	16, 17
	Varandei	Salix	68.657	58.375	0.72 [0.70,0.73]	0.57 [0.43,0.78]	22	38 (36-38)	16, 17
	Yuribei	Salix	68.837	70.322	0.63 [0.62,0.64]	0.29 [0.12,0.42]	28	74 (48-78)	16, 17
Sweden	Staloluokta	Salix	67.303	16.701	0.68 [0.66,0.69]	0.13 [-0.06,0.29]	27	17 (14-18)	15
USA	Arctic Alaska	Alnus	68.356	-159.914	0.38 [0.37,0.39]	0.73 [0.50,0.88]	15	90 (69-99)	15
	Noatak	Alnus	67.992	-162.003	0.54 [0.53,0.55]	0.52 [0.39,0.67]	22	65 (29-74)	20
	Sagwon_N	Alnus	69.016	-148.835	0.64 [0.62,0.65]	0.41 [0.18,0.63]	14	16 (9-17)	21
	Sagwon_N	Salix	69.016	-148.835	0.59 [0.56,0.62]	0.32 [0.04,0.54]	13	20 (18-20)	22
	Sagwon_S	Salix	68.729	-148.946	0.62 [0.61,0.64]	0.84 [0.72,0.93]	16	24 (9-28)	23

Supplementary Table 11 | Shrub sampling site locations, chronology inter-series correlations (rbar), sample sizes, and Spearman's correlations (r_s) between Landsat $NDVI_{max}$ and each shrub ring-width index chronology. Sample size includes the number of years of overlap between NDVI and shrub ring-width measurements (N Years) and the number of shrubs (N shrubs) that went into each chronology during these years. The number of shrubs in each chronology varied through time and thus the mean, min, and max sample sizes are provided. The rbar and r_s include 95% confidence intervals derived from 10^3 Monte Carlo simulations.

Data Source	Site	Lat.	Lon.	Years	$NDVI_{max}$	GPP	Ref.
AON	Cherskii	68.514	161.531	2014-2016	0.74 [0.74,0.75]	348 [319,391]	[24]
	Imnavait Fen	68.606	-149.311	2008-2018	0.64 [0.62,0.64]	209 [195,223]	[25]
	Imnavait Ridge	68.607	-149.296	2008-2018	0.60 [0.59,0.60]	186 [175,198]	[25]
	Imnavait Tussock	68.606	-149.304	2008-2018	0.65 [0.64,0.66]	210 [185,224]	[25]
FLUXNET	GL-NuF	64.131	-51.386	2008-2014	0.66 [0.66,0.67]	297 [268,312]	[26]
	GL-ZaF	74.481	-20.555	2008-2011	0.61 [0.61,0.61]	318 [243,441]	[27]
	GL-ZaH	74.473	-20.550	2000-2014	0.43 [0.42,0.43]	107 [98,114]	[28]
	RU-Che	68.613	161.341	2002-2005	0.63 [0.62,0.63]	160 [127,245]	[29]
	RU-Cok	70.829	147.494	2003-2013	0.66 [0.65,0.66]	343 [318,443]	[30]
	US-Atq	70.470	-157.409	2003-2008	0.58 [0.55,0.59]	198 [194,205]	[31]
	US-Ivo	68.487	-155.75	2004-2007	0.62 [0.62,0.64]	255 [236,296]	[32]

Supplementary Table 12 | Characteristics of flux tower sites used to evaluate the relationship between Landsat $NDVI_{max}$ (unitless) and ecosystem gross primary productivity (GPP; g C m^{-2} yr^{-1}). Estimates of median annual $NDVI_{max}$ and GPP are provided for each site with 95% confidence intervals derived from 10^3 Monte Carlo simulations.

Supplementary Methods

Landsat data sets, processing, and analyses

To characterize annual tundra greenness, we developed annual estimates of maximum summer normalized difference vegetation index ($NDVI_{max}$) from 1985 to 2016 for sampling sites in Arctic tundra[33] using 30 m resolution measurements of surface reflectance from the Landsat satellites (Landsat Collection 1)[34, 35]. Estimates of annual Landsat $NDVI_{max}$ are sensitive to multiple sources of uncertainty, including sensor calibration (± 3 to 7%)[36, 37], systematic differences in NDVI among sensors[38, 39], and the availability of summer measurements. We developed new approaches to cross-calibrate NDVI among sensors and model annual $NDVI_{max}$ using summer measurements in conjunction site-specific information on seasonal land surface phenology. Moreover, we ascertained how uncertainty in estimates of annual $NDVI_{max}$ time series affected subsequent aspects of the analysis using Monte Carlo simulations ($n = 10^3$). The following sections provide details regarding the Landsat data sets and processing.

Landsat data sets

We used measurements of land surface reflectance at 30 m resolution from 1985 to 2016 that were derived from Landsat 5, 7, and 8 by the United States Geological Survey (USGS) as part of the Landsat Collection 1 (Tier 1 and Tier 2) dataset. The USGS corrected Landsat 5 and 7 measurements for atmospheric and terrain effects using the Landsat Ecosystem Disturbance Adaptive Processing System[34] and corrected Landsat 8 measurements using the Landsat 8 Surface Reflectance Code[35]. Landsat 5 was operational from 1984 to 2013, while Landsat 7 and 8 have been operational from 1999 and 2013, respectively, to present. We accessed these Landsat data using the Python[40] interface for Google Earth Engine[41].

Landsat sampling

We extracted Landsat surface reflectance measurements for 50,000 terrestrial sampling sites spread randomly across the Arctic tundra. We included both Polar Arctic and Oro Arctic tundra[33] and partitioned terrestrial from aquatic areas using the Joint Research Center Global Surface Water dataset[42]. We buffered each sampling site by 50 m radius, yielding an approximate 3x3 pixel window around each site. For each pixel within a buffer we then extracted all Landsat 5, 7, and 8 surface reflectance measurements that were acquired June through August (Julian Days 152 – 243) between 1984 and 2016. This yielded 507 million multi-band measurements of land surface reflectance from these sampling sites. All together, we sampled 0.005% of the domain at an average density of one sampling site per 155 km^2 of land area. On average, sites had a nearest neighbor that was 7.0 km away (minimum = 47 m and maximum = 314 km). The adequacy of this sample size is justified below in the Landsat $NDVI_{max}$ trend analysis section.

Landsat quality control

We took multiple steps to ensure that only high-quality clear-sky measurements were included in our analysis. First, we exclude observations (i.e., a pixel at a point in time) from scenes with high cloud cover (> 80%), spatial uncertainty (> 30 m), or solar zenith angle (> 60°). Second, we masked out observations that were identified as cloud, cloud shadows, water, or snow by the C Function of Mask (CFmask) algorithm[43, 44]. Third, we minimized potential errors associated with radiometric saturation, atmospheric correction, or residual water by excluding observations with unrealistically high (> 1) or very low (< 0.005) surface reflectance. Fourth, we excluded

observations that fell within the data gaps caused by failure of the Landsat 7 scan line corrector. Overall, we filtered out 72% of observations due to these issues.

Cross-calibrating NDVI among Landsat sensors

There are systematic differences in NDVI among Landsat 5, 7, and 8 (Supplementary Figure 1) and these differences must be addressed before assessing temporal trends in NDVI[38, 39, 45]. Failure to address these differences can introduced artificial positive trends into NDVI time-series that are based on measurements from multiple Landsat sensors. Linear models have been developed to cross-calibrate Landsat 5 and 7 NDVI in boreal North America[38, 45] and Landsat 7 and 8 NDVI for the conterminous USA[39], but we are unaware of existing models for cross-calibrating Landsat 5, 7, and 8 in Arctic tundra. Following prior studies, we initially tried cross-calibrating NDVI among sensors using linear regression, but observed a nonlinear relationship between Landsat 7 and 8. We therefore developed a machine learning approach to cross-calibrate NDVI from Landsat 5 and 8 with Landsat 7, which is a useful benchmark since it temporally overlaps with the other sensors.

We cross-calibrated the sensors by first identifying the years when both Landsat 7 and Landsat 5/8 collected imagery at a sampling site. Second, we pooled NDVI measurements across those years and then computed 15-day moving median NDVI over the course of the growing season for each sensor and sampling site. Third, we excluded 15-day periods with fewer than 5 measurements from both sets of sensors and then randomly selected one remaining 15-day period from each sampling site. We then used 2/3^{rds} of these data to train Random Forest models[46] that predicted Landsat 7 NDVI based on Landsat 5/8 NDVI. The models also account for potential seasonal and regional differences between sensors by including as covariates the midpoint of each 15-day period (day of year) and the spatial coordinates of each sampling site. We fit the random forest models using the ranger package[47] in R[48]. As part of the Monte Carlo uncertainty analysis, we fit a separate random forest model to each of the 10^3 simulations.

In addition to the out-of-bag error assessment performed internally by the Random Forest, we also cross-validated each model using the remaining 1/3rd of the data that was withheld from training. This holdout cross-validation involved predicting NDVI using the trained Random Forest model and then linearly regressing observed versus predicted NDVI. The models for calibrating Landsat 5 and 8 had high predictive capacity ($r^2 \approx 0.97$) and both low root mean squared error and bias (

Supplementary Tables

Supplementary Figure 1). The performance of these models was similar to or exceeded that of cross-calibration models developed for other regions[39, 45, 49]. We therefore applied these models to cross-calibrate NDVI measurements at the full set of sampling sites.

Modeling maximum summer NDVI using Landsat

We sought to infer changes in tundra greenness using estimates of maximum summer NDVI ($NDVI_{max}$) derived from the Landsat satellites. It is challenging to reliably estimate annual $NDVI_{max}$ using Landsat since these estimates are sensitive to the number of cloud- and snow-free observations ("useable observations") acquired each summer. The annual number of useable summer observations increased from 1984 to 2016 at sites in the Arctic (Supplementary Figure 2). There were typically few useable summer observations at each site during the 1980s and 1990s, though observations became increasingly available during the 2000s following the launches of Landsat 7 and 8. Estimates of annual $NDVI_{max}$ typically increase asymptotically with the number of useable summer observations since this increases the likelihood that observations will have been acquired during peak summer greenness (Supplementary Figure 3). In other words, $NDVI_{max}$ is systematically underestimated when few observations are available. Consequently, the increase in useable summer observations introduces a spurious positive trend into $NDVI_{max}$ time series and this must be addressed before assessing long-term trends in $NDVI_{max}$. The irregular timing of image acquisitions and overall low image availability make it challenging to compute not only $NDVI_{max}$, but also time-averaged or integrated NDVI[50]. We therefore developed an approach to more reliably estimate $NDVI_{max}$ when few summer observations were available.

We estimated annual Landsat $NDVI_{max}$ at sampling sites by combining annual summer observations with information on land surface phenology. Land surface phenology can be characterized based on seasonal changes in NDVI. The NDVI typically increases in spring as snow melts and plants begin leafing-out, reaches a maximum in the middle of summer following full plant community canopy development, and then declines in fall as leaves senesce and are shed[51]. For each site we quantified land surface phenology from spring through fall by predicting daily NDVI using flexible cubic splines fit to all quality-controlled Landsat observations. To account for potential shifts in land surface phenology during recent decades[52, 53], we fit an individual cubic spline to observations from each 17-year period between 1985 and 2016 (Supplementary Figure 4). The Landsat record is limited in much of the Arctic prior to the 2000s, thus using a 17-year window allowed us to pool measurements across this era of sparse observations when estimating annual $NDVI_{max}$. As part of quality control, we iteratively removed observations with NDVI that differed by >100% from the corresponding daily prediction. We also excluded sites from 17-year periods if there were fewer than 30 useable observations. We fit the cubic splines using the smooth.spline function in R[48] and characterized uncertainty in model fit by randomly varying the smoothing parameter (spar =0.68 - 0.72) and available observations as part of the Monte Carlo analysis. We interpret each cubic spline as representing the typical land surface phenology of the site [54] during the corresponding 17-year period.

Landsat observations acquired during summer may not exactly coincide with the timing of peak summer greenness ($NDVI_{max}$); however, it is possible to estimate annual $NDVI_{max}$ by combining these summer observations with site-specific information on land surface phenology (example in Supplementary Figure 4b). The phenological curves enabled us to determine the typical difference in NDVI between peak summer greenness and the day that each summer

observation was acquired ($\Delta NDVI_{DOY}$, vertical blue lines in Supplementary Figure 4b). The $\Delta NDVI_{DOY}$ reflects the additional increase in NDVI that we would expect had a Landsat observation occurred during peak summer greenness instead of slightly earlier or later in the growing season. We then estimated annual $NDVI_{max}$ using each summer observation:

$$NDVI_{max} = NDVI_{DOY} + \Delta NDVI_{DOY} \quad (1)$$

where $NDVI_{DOY}$ is an observation of NDVI from a specific day during the summer. If multiple Landsat observation were available from a single summer, then we estimated overall $NDVI_{max}$ for that summer by computing the median of $NDVI_{max}$ predicted from each summer observation. While we focused on estimating inter-annual variability in $NDVI_{max}$, our approach is akin to one presented by Melass and colleagues[54, 55] who examined inter-annual variability in the start and end of the growing season in deciduous forest of eastern North America.

We evaluated how estimates of annual $NDVI_{max}$ changed with the availability of growing season scenes using both phenologically-corrected and raw (uncorrected) Landsat observations. Here we define the 'growing season' for each site using the phenological curves to identify the seasonal period when daily NDVI was typically within 75% of $NDVI_{max}$. We first selected site x years with at least 11 Landsat scenes acquired during a growing season. We then calculated observed $NDVI_{max}$ for each site x year; however, to guard against observations with spuriously high NDVI [51] we excluded the 10% of observations with the highest NDVI before computing $NDVI_{max}$. Next, we repeatedly subsampled between one and ten Landsat observations from these site x years and for each subsample computed how phenologically-corrected and raw estimates of $NDVI_{max}$ differed from observed $NDVI_{max}$. This allowed us to quantify how the percent error between observed and estimated $NDVI_{max}$ changed with scene availability both with and without our phenological correction (Supplementary Figure 3). This assessment showed that raw estimates of $NDVI_{max}$ increase asymptotically until there are at least seven Landsat scenes acquired during a summer, after which estimates of $NDVI_{max}$ change little with increasing scene availability. On the other hand, our phenologically-corrected estimates of $NDVI_{max}$ change little with increasing scene availability, though the uncertainty of the estimates decreases with increasing scene availability. Relative to raw data, the phenological correction slightly increased the spread of estimates when more than five scenes were available from a growing season, which occurred in about 9% of all site x years. These comparisons highlight that (1) estimates of annual $NDVI_{max}$ are sensitive to the number of available scenes and that (2) our phenological correction can provide less biased estimates of annual $NDVI_{max}$ when few Landsat scenes are available from a growing season.

Landsat $NDVI_{max}$ trend analysis

We assessed $NDVI_{max}$ trends during recent decades using Landsat observations from across the Arctic. Specifically, we evaluated $NDVI_{max}$ trends at individual sampling sites and after averaging $NDVI_{max}$ time series among sites within tundra bioclimatic zones and across the whole Arctic. Furthermore, we focused on $NDVI_{max}$ trends during two nominal periods (1985 to 2016 and 2000 to 2016) that were chosen based on (1) the availability of Landsat imagery in the Arctic and (2) interest in assessing both long-term and near-term trends. We excluded sampling sites that were barren (mean $NDVI_{max}$ < 0.10, n = 4,112 sites) or had short measurement records (< 10 years, n = 582 sites). We then evaluated each $NDVI_{max}$ time series for the presence of a monotonic trend using a rank-based Mann-Kendall trend test[56, 57] and determined the slope of

each time series using a non-parametric Theil-Sen slope estimator[58]. The Theil-Sen slope estimator and Mann-Kendall trend test were sequentially implemented by the zyp.yuepilon function from the zyp package[59] in R[48]. Temporal autocorrelation can inflate Mann-Kendall trend test statistics and increase the likelihood of detecting a trend when none is present[60] and thus the zyp.yuepilon function first evaluates whether a time series exhibits temporal autocorrelation and if temporal autocorrelation is identified then the time series is pre-whiten before implementing the Mann-Kendall trend test[60]. The Theil-Sen slope estimator and Mann-Kendall trend test are less sensitive to extreme values than simple linear regression and have been used in prior studies to assess NDVI trends at high-latitudes[61, 62]. The Landsat $NDVI_{max}$ trends are summarized for the Arctic and each bioclimatic zone in Supplementary Table 2 and Supplementary Table 3.

To evaluate the adequacy of our sample size, we examined how estimates of two trend metrics varied as a function of sample size. Focusing on 2000 to 2016, we computed the change in mean Arctic $NDVI_{max}$ and the percentage of sites with positive, negative, or no trend (α = 0.10) using sample sizes ranging from 10^2 to $4x10^4$ sites. Specifically, we used samples sizes from 10^2 to 10^3 sites at intervals of 10^2 sites and then from 10^3 to $4x10^4$ sites at intervals of 10^3 sites (n = 49 bins total). For each Monte Carlo simulation (n = 10^3), we computed these trend metrics using random subsets of sites for each of the 49 sample size bins. We then computed the median and 95% confidence interval (CI) of each trend metric for every sample size bin. This analysis revealed minimal differences in trend estimates across a 400-fold range in sample size (Supplementary Table 4, Supplementary Figure 5). For instance, we estimated that the median increases in mean Arctic $NDVI_{max}$ was 3.51%, 3.38%, or 3.35% whether based on 10^2, 10^4, or $4x10^4$ sample sites. Moreover, we estimated that the median percentage of sites with a positive $NDVI_{max}$ trend (α = 0.10; greening) was 21.00%, 21.30%, and 21.30% based on 10^2, 10^4, or $4x10^4$ sampling sites, while the median percentage of sites with a negative $NDVI_{max}$ trend (browning) was 6.00% across all sizes. The width of the 95% CIs associated with these trend metrics asymptotically shrank lead to about a 0.5% change between 10^4 and $4x10^4$ sampling sites (Supplementary Figure 5c,d). This analysis illustrates the sample size is adequate for drawing robust inference about recent changes in tundra greenness across the Arctic.

Air temperature data sets, processing, and analyses

We acquired and pre-processed five global gridded temperature data sets (Supplementary Table 5) and then, for the Arctic region, derived the summer warmth index (SWI) as a metric of cumulative summer heat load[63]. Three data sets provided estimates of monthly mean air temperature (T_{avg}; °C) and two data sets provided estimates of monthly T_{avg} anomaly relative to a climatological baseline (1951-1980 for NASA GISS and 1981-2010 for UY HadCRUT4 with UAH). Each data set was publicly available online and provided data for at least the period from 1979 to 2016. We clipped data sets to the Arctic domain[33] and projected each to Lambert Azimuthal Equal Area on a 50-km grid using bilinear interpolation. For the two monthly T_{avg} anomaly data sets, we estimated absolute monthly T_{avg} by adding a monthly climatological baseline derived from the ensemble average of the three absolute T_{avg} data sets. We then derived and applied a common mask that only kept grid cells with non-missing data from every data set. Next, we computed the annual SWI as the sum of monthly T_{avg} exceeding 0 °C. The SWI is commonly used as an indicator of cumulative heat load in the Arctic[49, 63, 64] and is analogous to growing-degree days, but computed using monthly rather than daily temperature data.

Each temperature dataset was constructed using different collections of climate station observations and analytical techniques[1, 2, 4, 5, 6], thus temporal trends in SWI and correlations between SWI and $NDVI_{max}$ are influenced by the specific temperature data set used in the analysis. To account for uncertainty in trends and correlation stemming from the climate data sets, we generated 10^3 synthetic domain-wide rasters of SWI for each year from 1984 to 2016. For each grid cell of every synthetic raster, we assigned a value for SWI that was randomly selected from the corresponding grid cell of one of the five temperature data sets. Consequently, each synthetic raster was built using a randomized assortment of grid cell values from the five temperature data sets. We then used this collection of synthetic SWI rasters to assess temporal trends in SWI as well as correlation between SWI and $NDVI_{max}$ (as described below).

Trends in summer air temperatures

We assessed changes in Arctic summer temperatures using the synthetic SWI raster data sets (described above) and non-parametric trend tests in a Monte Carlo uncertainty framework. Specifically, for each of the 10^3 synthetic SWI data sets, we evaluated SWI trends from 1985 to 2016 and 2000 to 2016 using non-parametric Mann-Kendall and Theil-Sen tests as implemented by the zyp.yuepilon function from the zyp package[59] in R[48]. We assessed SWI trends for each grid cell and Landsat sampling site, as well as after averaging SWI among grid cells in each bioclimatic zone and across the Arctic domain. We report the median change across all simulations as our best estimate of each trend and a 95% confidence interval computed from the 2.5^{th} and 97.5^{th} percentiles of these simulations. Changes mean SWI for the Arctic and each bioclimatic zone are given in Supplementary Table 6.

Temporal correspondence between Landsat $NDVI_{max}$ and summer temperatures

We assessed the temporal correspondence between annual Landsat $NDVI_{max}$ and summer air temperatures (SWI) from 1985 to 2016 and 2000 to 2016 at multiple spatial scales. We evaluated the direction and strength of correspondence between $NDVI_{max}$ and SWI using rank-based Spearman's correlations (r_s) in a Monte Carlo uncertainty framework. Specifically, we computed $NDVI_{max}$ - SWI correlations for individual sampling sites and after averaging mean-centered $NDVI_{max}$ and SWI time series among sites within tundra bioclimatic zones and across the Arctic. Tundra greenness ($NDVI_{max}$) could depend on summer temperatures over multiple years so we correlated $NDVI_{max}$ with current and 2-year average SWI. The strength of $NDVI_{max}$ - SWI correlations could also be influenced by underlying trends in both time series (e.g., warming and greening) thus we derived correlations using both original and linearly detrended time series. Moreover, uncertainty in estimates of $NDVI_{max}$ and SWI could influence their temporal covariation. We therefore derived 10^3 simulations of every correlation, with each simulation based on randomly permutated estimates of $NDVI_{max}$ and SWI. We present the median r_s of all simulations as our best estimate for each $NDVI_{max}$ - SWI correlation and report a 95% confidence interval derived from the 2.5^{th} and 97.5^{th} percentile of all r_s simulations. The $NDVI_{max}$ – SWI correlations for each zone are summarized in Supplementary Table **7** while spatial patterns of these correlations are summarized in Supplementary Figure 6.

Comparisons among Landsat $NDVI_{max}$ and plant productivity measurements

We assessed the utility of Landsat $NDVI_{max}$ as an indicator of tundra plant productivity using field measurements from across the Arctic. We compared Landsat $NDVI_{max}$ against measurements of graminoid aboveground net primary productivity (ANPP; dry matter m^{-2} yr^{-1}) and shrub ring-width indices (RWI; unitless), as well as estimates of ecosystem gross primary productivity (GPP; g C m^{-2} yr^{-1}) derived from flux towers. We describe the data sets and specific comparisons in greater detail below, but in each case, we assessed the direction and strength of association between satellite and field measurements using rank-based Spearman's correlations (r_s) evaluated in a Monte Carlo framework that incorporated uncertainty in $NDVI_{max}$ and field measurements. Together, these field datasets span six countries (Canada, Finland, Greenland, Russia, Sweden, and USA) and several important plant functional types in the tundra biome (Supplementary Figure **7**).

Landsat $NDVI_{max}$ vs. graminoid productivity

We assessed the temporal correspondence between annual Landsat $NDVI_{max}$ and graminoid ANPP from 1990 to 2017 on Bylot Island in northern Canada (Supplementary Figure **7**a)[65]. Graminoid ANPP has been measured since 1990 in a moss-covered wetland fen that is dominated by grasses and sedges (e.g., *Dupontia fisheri*, *Carex aquatilis*, *Eriophorum Scheuchzeri*). This long-term monitoring is part of a project focused on Arctic food chains and provides, to our knowledge, the longest annual record of plant productivity in the tundra biome[65, 66, 67]. The long record and spatially-extensive sampling during peak summer make these field data particularly valuable for evaluating remote sensing indicators of plant productivity[68].

Graminoid ANPP was quantified each year by clip harvesting live graminoid aboveground biomass (AGB) from quadrats (20 x 20 cm) that were randomly positioned across several subsites in the wetland. The harvests occurred when graminoid AGB reached a maximum in mid-August (Julian day = 226 ± 2 days; mean ± SD) and thus provide an estimate of ANPP given annual turn-over of graminoid AGB[66]. There were typically 12 quadrats harvested per year (but 11 quadrats in 1991, 2013, 2014, and 2016), with six quadrats harvested at each of two subsites (n = 332 quadrats total across years). One subsite was continually measured over the 28-year period; however, the second subsite was measured once in 1990, moved to a nearby location for measurements in 1991 and 1992, and then moved again in 1993 after which the location remained the same.

We examined the temporal correspondence between annual median landscape Landsat $NDVI_{max}$ and graminoid ANPP from 1990 to 2017 using Spearman's correlations (r_s) in a Monte Carlo uncertainty framework ($n = 10^3$ simulations). This involved extracting and quality-screening all Landsat summer observations from a 100 m radius area around each subsite and then averaging spectral measurements from each Landsat scene. For each simulation, we first estimated annual median $NDVI_{max}$ across subsites after (1) computing NDVI with randomly permuting red and NIR reflectance, (2) cross-calibrating NDVI among sensors using a randomly selected Random Forest model and (3) fitting phenological curves with randomly permuted parameters. Second, we estimated annual median ANPP using data from a random subset (90%) of quadrats each year. Lastly for each simulation, we computed the correlation between annual median $NDVI_{max}$ and ANPP using data from a random subset (90%) of years from 1990 to 2017. The annual median ANPP time series was temporally autocorrelated over a two-year period ($r_{lag1} = 0.64$ and $r_{lag2} = 0.66$, $P < 0.05$) and thus we chose to compare annual median ANPP with $NDVI_{max}$ from not only the concurrent year, but also averaged over the current and two prior years, as well as just the two prior years. We present the median r_s as our best estimate of each

correlation and a 95% confidence interval derived from the 2.5th and 97.5th percentiles of the 10^3 Monte Carlo simulations.

Landsat $NDVI_{max}$ vs. shrub growth

We assessed the temporal correspondence between annual Landsat $NDVI_{max}$ and 22 shrub ring-width index (RWI) chronologies from sites located in six Arctic countries (Supplementary Figure 7b). These shrub RWI chronologies are a proxy for interannual variability in shrub productivity and in some cases may co-vary with broader plant community productivity[69]. We used new and archived annual ring-width measurements from independent projects[16, 17, 21, 22, 23, 70, 71], including measurements previously collated as part of the ShrubHub shrub ring database[15]. The data set included annual ring-width measurements for alder (*Alnus* spp.), willow (*Salix* spp.), and birch (*Betula* spp.), which are genera of tall deciduous shrubs that are widespread across much of the circumpolar Oro Arctic and Low Arctic[72].

Sample collection and measurement protocols differed among projects, but each project ultimately generated annual shrub ring-width measurements. Sampling occurred between c. 2005 and 2017, and typically involved harvesting the largest shrubs found in a study area, though one project harvested shrubs along transects[71]. Several projects recorded the location of every stem or transect sampled in a study area[16, 17], whereas most recorded one or several general sampling locations. Shrubs were harvested near the root collar and then one or more discs was cut from the bottom of each stem. Each disc was sanded, some discs were stained, and then the width of each growth ring was measured along one or multiple radii using either a stereo-microscope and sliding stage or a digital camera and imaging software. Each ring-width series was then cross-dated to assure that growth rings were ascribed the proper calendar year. Additional details regarding sample collection and measurement protocols can be found in the references cited above. Overall, we drew on 54,374 cross-dated measurements of annual ring-widths from 1,348 shrubs (17 to 297 shrubs per study area).

We constructed median shrub ring-width index (RWI) chronologies for each shrub genera at every sampling site (n = 22 chronologies). First, we minimized the effects of juvenile growth by excluding the initial five years of measurements[16] and then averaged ring-width measurements made along multiple radii of an individual shrub. Second, we removed potential age-related biological growth trends and standardized the magnitude of growth among individual shrubs. This was accomplished by fitting a flexible cubic spline to each time series and then dividing observed ring-width in year t by the ring-width predicted for year t by the spline[73]. We fit each spline using the ffcsaps function from the dendrochronological program library in R (dplR)[74]. To account for uncertainty in this process, we randomly varied spline flexibility and moving-window length as each spline was fit. Consequently, each spline removed 45-55% of the variance over a 15-25 year moving window, thus preserving high-frequency interannual fluctuations in growth while removing low-frequency (i.e., multi-decadal) growth trends. Lastly, for each simulation we generated a genus-specific shrub RWI chronology at each sampling site by computing annual median RWI using data from a random subsample (90%) of shrubs.

We also constructed annual Landsat $NDVI_{max}$ time series to compare against the shrub RWI chronologies. This first involved extracting and quality-screening all Landsat summer observations from a 100 m radius area around each geotagged sampling location in a study area. For each simulation, we estimated annual $NDVI_{max}$ for every sampling location by computing NDVI with randomly permuting red and NIR reflectance, (2) cross-calibrating NDVI among sensors using a randomly selected random forest model and (3) fitting phenological curves with

randomly permuted parameters. We then detrended the annual $NDVI_{max}$ ($NDVI_{max-dt}$) timeseries for each sampling location using flexible splines (as above) and computed annual median $NDVI_{max-dt}$ across sampling locations in each study area. Lastly, for each simulation we computed the Spearman correlations (r_s) between annual median $NDVI_{max-dt}$ and each shrub RWI chronology. We present the median r_s for each site as our best estimate of the relationship and a 95% confidence interval derived from the 2.5th and 97.5th percentiles of the 10^3 Monte Carlo simulations.

Landsat $NDVI_{max}$ vs. ecosystem productivity

We assessed the spatial correspondence between median annual Landsat $NDVI_{max}$ and ecosystem GPP across 11 flux towers located in Arctic tundra of Greenland, Russia, and the USA (Supplementary Figure **7**, Supplementary Table 12). Four of the flux towers were part of the Arctic Observing Network (AON)[24, 25] and seven of the flux towers were part of the FLUXNET Network (FLUXNET2015 CC-BY-4.0 February 2020)[75]. The net land-atmosphere CO_2 exchange (i.e., net ecosystem exchange [NEE]) was measured at each site using the eddy covariance technique, which involves coupling measurements of atmospheric CO_2 concentrations and meteorological conditions from instruments mounted on towers. Both AON and FLUXNET then estimated GPP and ecosystem respiration (R_{eco}) by partitioning NEE (NEE = GPP – R_{eco}) using modeled relationships between R_{eco} and nighttime temperatures[76]. We acquired annual gap-filled estimates of GPP from FLUXNET and half-hourly gap-filled estimates of GPP from AON that we aggregated to an annual time step (g C m^{-2} yr^{-1}).

We assessed the spatial correspondence between median annual $NDVI_{max}$ and GPP across 11 flux tower sites using Spearman's correlations (r_s) in a Monte Carlo uncertainty framework (n = 10^3 simulations). We chose to examine the relationship across rather than within sites because the annual GPP time series at each site was relatively short (mean = 7.7 years, SD = 3.7 years). Each simulation involved randomly permuting $NDVI_{max}$ and GPP data sets before computing the correlation between these metrics.

To propagate uncertainty in annual estimates of GPP into our analysis, we constructed distributions of annual GPP for each site x year and then randomly drew from these distributions during each simulation. The FLUXNET data included uncertainty estimates for annual GPP at each site that were provided as the 5th, 16th, 25th, 50th, 75th, 84th, and 95th distribution percentiles of a bootstrap analysis that applied varying friction velocity (u*) thresholds to delineate well mixed from poorly mixed atmospheric conditions. We constructed a distribution of annual GPP for each site x year by linearly interpolating between the distribution percentiles provided with the data set. The AON data set relied on a fixed u* threshold (0.1 m s^{-1}) and did not include uncertainty estimates, thus we constructed a distribution of annual GPP for each site x year by relying on uncertainty derived from the FLUXNET data. Specifically, for each FLUXNET site x year we computed the ratios of GPP at the 50th percentile to GPP at every other percentile ('uncertainty fraction) and then computed the median uncertainty fraction for each percentile across all site x years. We then generated synthetic distributions of annual GPP for each AON site x year by multiplying annual GPP by the vector of median uncertainty fractions. Overall, this process yielded distributions of annual GPP for every site x year in both the FLUXNET and AON data sets.

To propagate uncertainty in annual estimates of $NDVI_{max}$ into our analysis, we generated 10^3 time series of annual $NDVI_{max}$ from 1985 to 2017 for each flux tower site. We first extracted and quality-screened all Landsat summer observations from a 100 m radius area around each flux

tower site and then averaged spectral measurements from each Landsat scene. Next, we estimated annual $NDVI_{max}$ for each site by (1) computing NDVI with randomly permuting red and NIR reflectance, (2) cross-calibrating NDVI among sensors using a randomly selected random forest model and (3) fitting phenological curves with randomly permuted parameters.

For each simulation, we computed median annual $NDVI_{max}$ and GPP by site using data from a random subset (90%) of years and annual GPP randomly drawn from its corresponding distribution. We then computed the correlation between median annual $NDVI_{max}$ and GPP. We present the median r_s as our best estimate of the relationship and a 95% confidence interval derived from the 2.5^{th} and 97.5^{th} percentiles of the 10^3 Monte Carlo simulations.

Supplementary Discussion

Relationship between Landsat $NDVI_{max}$ and graminoid productivity

We found that inter-annual variability in Landsat $NDVI_{max}$ and graminoid ANPP were positively correlated at a long-term field monitoring site in northern Canada. Furthermore, we found that annual graminoid ANPP was autocorrelated over the preceding two years and that the $NDVI_{max}$ – ANPP relationship was strongest if $NDVI_{max}$ was averaged over the preceding two years. These findings suggest that annual graminoid ANPP partially depends on conditions during previous growing seasons. The lagged relationships could reflect the importance of non-structural carbohydrates and nutrients acquired in previous years, which are temporarily stored in below-ground tissues (e.g., rhizomes) and later used for biosynthesis[77]. Arctic graminoids typically have a high ratio of belowground to aboveground biomass (e.g., the ratio for *Carex aquatilis* reportedly ranges from 3.4 to 22.6)[78] underscoring the importance of below-ground processes in these ecosystems. Although based on observations from one study area, the positive correlation between Landsat $NDVI_{max}$ and graminoid ANPP provides support for interpreting Landsat $NDVI_{max}$ as a proxy for aspects of tundra plant productivity associated with graminoids.

Relationship between Landsat NDVImax and ecosystem productivity

We found that median annual Landsat $NDVI_{max}$ was related to broad spatial patterns of median annual tundra ecosystem GPP. Our remote sensing analysis builds on prior studies that showed positive associations between hand-held measurements of NDVI and short-term, chamber-based estimates of tundra ecosystem GPP[79, 80]. The relationship between NDVI and GPP likely arises because NDVI is a proxy for leaf area and chlorophyll content which together influence canopy light absorption and subsequent GPP[80, 81]. Overall, these results provide support for interpreting Landsat $NDVI_{max}$ as a proxy for GPP in tundra ecosystems.

Relationship between Landsat NDVImax and shrub growth

Our assessment revealed weak to moderate positive correspondence between interannual variability in Landsat $NDVI_{max}$ and shrub radial growth. Prior efforts have similarly demonstrated modest positive correspondence between AVHRR NDVI and both shrub[16, 17, 82] and tree[62, 83, 84] radial growth in northern ecosystems. To our knowledge, Landsat NDVI has not previously been compared with interannual variability in shrub radial growth, but the generally positive but modest correspondence is not entirely surprising since both are metrics of carbon exchange. The NDVI is related to plant canopy leaf area and nitrogen content that affect light harvesting and subsequent carbon uptake (GPP) by the plant community[80, 81]. On the other hand,

shrub radial growth reflects carbon assimilation into plant aboveground woody tissues, which is but one aspect of whole plant NPP that also includes leaf and belowground productivity. Consequently, NDVI and radial growth are imperfect proxies for different metrics of carbon exchange that we might expect to positively covary under the assumption that both reflect inter-annual variations between better and worse years of vegetation growth[84].

The degree of covariation between these proxies will likely be affected not only by interannual variability in respiration and allocation, but also by landscape heterogeneity [84]. Shrubs can have a strong effect on NDVI in tundra ecosystems[16, 49, 85], but are one function type of varying dominance in overall plant communities[49, 86], which are themselves embedded in a mosaic of different land cover types. Consequently, we hypothesize that the correspondence between NDVI and shrub RWI is probably related to plant community and land cover heterogeneity, and the degree to which shrub RWI reflects fluctuations in above-ground plant productivity across the landscape[87]. This topic deserves future attention. Overall, the modest positive correspondence between Landsat $NDVI_{max}$ and shrub radial growth provides support for interpreting Landsat $NDVI_{max}$ as a proxy for aspects of tundra plant productivity associated with shrubs.

Supplementary References

1. Rohde R*, et al.* A new estimate of the average Earth surface land temperature spanning 1753 to 2011. *Geoinformatics and Geostatistics: An Overview* **7**, 10.4172/2327-4581.1000101 (2013).

2. Cowtan K, Way RG. Coverage bias in the HadCRUT4 temperature series and its impact on recent temperature trends. *Quarterly Journal of the Royal Meteorological Society* **140**, 1935-1944 (2014).

3. GISTEMP Team. *GISS Surface Temperature Analysis (GISTEMP)*. NASA Goddard Institute for Space Studies, https://data.giss.nasa.gov/gistemp/ (2019).

4. Hansen J, Ruedy R, Sato M, Lo K. Global surface temperature change. *Reviews of Geophysics* **48**, RG4004 (2010).

5. Harris I, Jones PD, Osborn TJ, Lister DH. Updated high-resolution grids of monthly climatic observations – the CRU TS3.10 Dataset. *International Journal of Climatology* **34**, 623-642 (2014).

6. Willmott CJ, Matsuura K. *Terrestrial Air Temperature and Precipitation: Monthly Time Series (1900 - 2017) v. 5.01*. University of Deleware, http://climate.geog.udel.edu/~climate (2018).

7. Abatzoglou JT, Dobrowski SZ, Parks SA, Hegewisch KC. TerraClimate, a high-resolution global dataset of monthly climate and climatic water balance from 1958–2015. *Scientific Data* **5**, 170191 (2018).

8. Obu J*, et al. ESA Permafrost Climate Change Initiative (Permafrost_cci): Permafrost Active Layer Thickness for the Northern Hemisphere, v1.0*. Centre for Environmental Data Analysis, 10.5285/1ee56c42cf6c4ef698693e00a63795f4 (2019).

9. Obu J*, et al. ESA Permafrost Climate Change Initiative (Permafrost_cci): Permafrost Ground Temperature for the Northern Hemisphere, v1.0.* Centre for Environmental Data Analysis, 10.5285/c7590fe40d8e44169d511c70a60ccbcc (2019).

10. Obu J*, et al. ESA Permafrost Climate Change Initiative (Permafrost_cci): Permafrost Extent for the Northern Hemisphere, v1.0.* Centre for Environmental Data Analysis, 10.5285/c7590fe40d8e44169d511c70a60ccbcc (2019).

11. Olefeldt D*, et al. Arctic Circumpolar Distribution and Soil Carbon of Thermokarst Landscapes, 2015*. ORNL DAAC, 10.3334/ORNLDAAC/1332 (2015).

12. Giglio L, Boschetti L, Roy DP, Humber ML, Justice CO. The Collection 6 MODIS burned area mapping algorithm and product. *Remote Sensing of Environment* **217**, 72-85 (2018).

13. Rizzoli P*, et al.* Generation and performance assessment of the global TanDEM-X digital elevation model. *ISPRS Journal of Photogrammetry and Remote Sensing* **132**, 119-139 (2017).

14. Defourny P*, et al. Land Cover Climate Change Initiative - Product User Guide Version v2*. European Space Agency, http://maps.elie.ucl.ac.be/CCI/viewer/download/ESACCI-LC-Ph2-PUGv2_2.0.pdf (2017).

15. Myers-Smith IH*, et al.* Climate sensitivity of shrub growth across the tundra biome. *Nature Climate Change* **5**, 887-891 (2015).

16. Forbes BC, Fauria MM, Zetterberg P. Russian Arctic warming and 'greening' are closely tracked by tundra shrub willows. *Global Change Biology* **16**, 1542-1554 (2010).

17. Macias-Fauria M, Forbes BC, Zetterberg P, Kumpula T. Eurasian Arctic greening reveals teleconnections and the potential for structurally novel ecosystems. *Nature Climate Change* **2**, 613-618 (2012).

18. Sullivan P. *Betula nana ring widths*. Arctic Data Center, 10.18739/A28Q18 (2016).

19. Sullivan P. *Salix glauca ring width*. Arctic Data Center, 10.18739/A24X0Q (2016).

20. Gaglioti B*, et al.* Tussocks enduring or shrubs greening: Alternate responses to changing fire regimes in the Noatak River Valley, Alaska. *Journal of Geophysical Research: Biogeosciences*, (Submitted).

21. Andreu-Hayles L, Anchukaitis K, D'Arrigo R, Martin-Fernandez J. *Shrub ring-width measurements of Alnus viridis var. crispa located on a slope over a small hill near the 'Ice Cut' at Dalton Highway in Alaska, 1920 - 2010*. National Center for Environmental Information, https://www.ncdc.noaa.gov/paleo/study/29752 (2020).

22. Andreu-Hayles L, Anchukaitis K, D'Arrigo R, Martin-Fernandez J. *Shrub ring-width measurements of Salix species over flat terrain next to the Sag River near the 'Ice Cut' at Dalton Highway in Alaska, 1977- 2010*. National Center for Environmental Information, https://www.ncdc.noaa.gov/paleo/study/29753 (2020).

23. Andreu-Hayles L, Anchukaitis K, D'Arrigo R, Martin-Fernandez J. *Shrub ring-width measurements of Salix species over flat terrain near the Dalton Highway in Alaska, 1964 - 2010*. National Center for Environmental Information, https://www.ncdc.noaa.gov/paleo/study/29754 (2020).

24. Euskirchen ES, Edgar CW, Syndonia Bret-Harte M, Kade A, Zimov N, Zimov S. Interannual and Seasonal Patterns of Carbon Dioxide, Water, and Energy Fluxes From Ecotonal and Thermokarst-Impacted Ecosystems on Carbon-Rich Permafrost Soils in Northeastern Siberia. *Journal of Geophysical Research: Biogeosciences* **122**, 2651-2668 (2017).

25. Euskirchen E, Bret-Harte MS, Scott G, Edgar C, Shaver GR. Seasonal patterns of carbon dioxide and water fluxes in three representative tundra ecosystems in northern Alaska. *Ecosphere* **3**, 10.1890/ES1811-00202.00201 (2012).

26. Hansen BU. *(2008-2014) FLUXNET2015 GL-NuF Nuuk Fen Dataset*. FLUXNET, https://doi.org/10.18140/FLX/1440222.

27. Lund M, Jackowicz-Korczyński M, Abermann J. *(2008-2011) FLUXNET2015 GL-ZaF Zackenberg Fen Dataset*. FLUXNET, https://doi.org/10.18140/FLX/1440223.

28. Lund M, Jackowicz-Korczyński M, Abermann J. *(2000-2014) FLUXNET2015 GL-ZaH Zackenberg Heath Dataset*. FLUXNET, https://doi.org/10.18140/FLX/1440224.

29. Merbold L, Rebmann C, Corradi C. *(2002-2005) FLUXNET2015 RU-Che Cherski Dataset*. FLUXNET, https://doi.org/10.18140/FLX/1440181.

30. Dolman H*, et al. (2003-2014) FLUXNET2015 RU-Cok Chokurdakh Dataset*. FLUXNET, https://doi.org/10.18140/FLX/1440182.

31. Zona D, Oechel W. *(2003-2008) FLUXNET2015 US-Atq Atqasuk Dataset*. FLUXNET, https://doi.org/10.18140/FLX/1440067.

32. Zona D, Oechel W. *(2004-2007) FLUXNET2015 US-Ivo Ivotuk Dataset*. FLUXNET, https://doi.org/10.18140/FLX/1440073.

33. Virtanen R*, et al.* Where do the treeless tundra areas of northern highlands fit in the global biome system: toward an ecologically natural subdivision of the tundra biome. *Ecology and Evolution* **6**, 143-158 (2016).

34. Masek JG*, et al.* A Landsat surface reflectance dataset for North America, 1990-2000. *IEEE Geoscience and Remote Sensing Letters* **3**, 68-72 (2006).

35. Vermote E, Justice C, Claverie M, Franch B. Preliminary analysis of the performance of the Landsat 8/OLI land surface reflectance product. *Remote Sensing of Environment* **185**, 46-56 (2016).

36. Markham BL, Helder DL. Forty-year calibrated record of earth-reflected radiance from Landsat: A review. *Remote Sensing of Environment* **122**, 30-40 (2012).

37. Markham B, *et al.* Landsat-8 operational land imager radiometric calibration and stability. *Remote Sensing* **6**, 12275-12308 (2014).

38. Ju J, Masek JG. The vegetation greenness trend in Canada and US Alaska from 1984–2012 Landsat data. *Remote Sensing of Environment* **176**, 1-16 (2016).

39. Roy DP, *et al.* Characterization of Landsat-7 to Landsat-8 reflective wavelength and normalized difference vegetation index continuity. *Remote Sensing of Environment* **185**, 57-70 (2016).

40. Python Software Foundation. *Python Language Software version 3.7.3.* https://www.python.org/ (2020).

41. Gorelick N, Hancher M, Dixon M, Ilyushchenko S, Thau D, Moore R. Google Earth Engine: Planetary-scale geospatial analysis for everyone. *Remote Sensing of Environment* **202**, 18-27 (2017).

42. Pekel J-F, Cottam A, Gorelick N, Belward AS. High-resolution mapping of global surface water and its long-term changes. *Nature* **540**, 418-422 (2016).

43. Foga S, *et al.* Cloud detection algorithm comparison and validation for operational Landsat data products. *Remote Sensing of Environment* **194**, 379-390 (2017).

44. Zhu Z, Wang S, Woodcock CE. Improvement and expansion of the Fmask algorithm: cloud, cloud shadow, and snow detection for Landsats 4–7, 8, and Sentinel 2 images. *Remote Sensing of Environment* **159**, 269-277 (2015).

45. Sulla-Menashe D, Friedl MA, Woodcock CE. Sources of bias and variability in long-term Landsat time series over Canadian boreal forests. *Remote Sensing of Environment* **177**, 206-219 (2016).

46. Liaw A, Wiener M. Classification and Regression by randomForest. *R News* **2**, 18-22 (2002).

47. Wright MN, Ziegler A. Ranger: a fast implementation of random forests for high dimensional data in C++ and R. *Journal of Statistical Software* **77**, 1-17 (2017).

48. R Core Team. *R: A Language and Environment for Statistical Computing*. R Foundation for Statistical Computing (2020).

49. Berner LT, Jantz P, Tape KD, Goetz SJ. Tundra plant aboveground biomass and shrub dominance mapped across the North Slope of Alaska. *Environmental Research Letters* **13**, 035002 (2018).

50. Karlsen SR, Anderson HB, Van der Wal R, Hansen BB. A new NDVI measure that overcomes data sparsity in cloud-covered regions predicts annual variation in ground-based estimates of high arctic plant productivity. *Environmental Research Letters* **13**, 025011 (2018).

51. Pettorelli N, Vik JO, Mysterud A, Gaillard J-M, Tucker CJ, Stenseth NC. Using the satellite-derived NDVI to assess ecological responses to environmental change. *Trends in Ecology & Evolution* **20**, 503-510 (2005).

52. Park T*, et al.* Changes in timing of seasonal peak photosynthetic activity in northern ecosystems. *Global Change Biology* **25**, 2382-2395 (2019).

53. Goetz SJ, Bunn AG, Fiske GJ, Houghton RA. Satellite-observed photosynthetic trends across boreal North America associated with climate and fire disturbance. *Proceedings of the National Academy of Sciences of the United States of America* **102**, 13521-13525 (2005).

54. Melaas EK*, et al.* Multisite analysis of land surface phenology in North American temperate and boreal deciduous forests from Landsat. *Remote Sensing of Environment* **186**, 452-464 (2016).

55. Melaas EK, Friedl MA, Zhu Z. Detecting interannual variation in deciduous broadleaf forest phenology using Landsat TM/ETM+ data. *Remote Sensing of Environment* **132**, 176-185 (2013).

56. Mann HB. Nonparametric tests against trend. *Econometrica* **13**, 245-259 (1945).

57. Kendall MG. *Rank Correlation Methods*, 4th edn. Charles Griffin (1975).

58. Sen PK. Estimates of the regression coefficient based on Kendall's tau. *Journal of the American Statistical Association* **63**, 1379-1389 (1968).

59. Bronaugh D, Werner A. *zyp: Zhang + Yue-Pilon trends package. R package version 0.10-1.1.* https://CRAN.R-project.org/package=zyp (2012).

60. Yue S, Pilon P, Phinney B, Cavadias G. The influence of autocorrelation on the ability to detect trend in hydrological series. *Hydrological processes* **16**, 1807-1829 (2002).

61. Guay KC, Beck PSA, Berner LT, Goetz SJ, Baccini A, Buermann W. Vegetation productivity patterns at high northern latitudes: a multi-sensor satellite data assessment. *Global Change Biology* **20**, 3147-3158 (2014).

62. Berner LT, Beck PSA, Bunn AG, Goetz SJ. Plant response to climate change along the forest-tundra ecotone in northeastern Siberia. *Global Change Biology* **19**, 3449-3462 (2013).

63. Walker D*, et al.* Phytomass, LAI, and NDVI in northern Alaska: relationships to summer warmth, soil pH, plant functional types, and extrapolation to the circumpolar Arctic. *Journal of Geophysical Research* **108**, 8169 (2003).

64. Bhatt US*, et al.* Changing seasonality of panarctic tundra vegetation in relationship to climatic variables. *Environmental Research Letters* **12**, 1-18 (2017).

65. Gauthier G*, et al.* Long-term monitoring at multiple trophic levels suggests heterogeneity in responses to climate change in the Canadian Arctic tundra. *Phil Trans R Soc B* **368**, 20120482 (2013).

66. Gauthier G, Hughes RJ, Reed A, Beaulieu J, Rochefort L. Effect of grazing by greater snow geese on the production of graminoids at an arctic site (Bylot Island, NWT, Canada). *Journal of Ecology* **83**, 653-664 (1995).

67. Legagneux P*, et al.* Disentangling trophic relationships in a High Arctic tundra ecosystem through food web modeling. *Ecology* **93**, 1707-1716 (2012).

68. Doiron M, Legagneux P, Gauthier G, Lévesque E. Broad-scale satellite Normalized Difference Vegetation Index data predict plant biomass and peak date of nitrogen concentration in Arctic tundra vegetation. *Applied Vegetation Science* **16**, 343-351 (2013).

69. Le Moullec M, Buchwal A, Wal R, Sandal L, Hansen BB. Annual ring growth of a widespread high arctic shrub reflects past fluctuations in community-level plant biomass. *Journal of Ecology* **107**, 436–451 (2019).

70. Tape KD, Hallinger M, Welker JM, Ruess RW. Landscape heterogeneity of shrub expansion in Arctic Alaska. *Ecosystems* **15**, 711-724 (2012).

71. Gamm CM*, et al.* Declining growth of deciduous shrubs in the warming climate of continental western Greenland. *Journal of Ecology* **106**, 640-654 (2018).

72. Walker DA*, et al.* The Circumpolar Arctic vegetation map. *Journal of Vegetation Science* **16**, 267-282 (2005).

73. Cook ER, Peters K. The smoothing spline: a new approach to standardize on forest interior tree-ring width series dendroclimatic studies. *Tree-Ring Bulletin* **41**, 45-52 (1981).

74. Bunn AG. A dendrochronology program library in R (dplR). *Dendrochronologia* **26**, 115-124 (2008).

75. Baldocchi D*, et al.* FLUXNET: A new tool to study the temporal and spatial variability of ecosystem-scale carbon dioxide, water vapor, and energy flux densities. *Bulletin of the American Meteorological Society* **82**, 2415-2434 (2001).

76. Reichstein M*, et al.* On the separation of net ecosystem exchange into assimilation and ecosystem respiration: review and improved algorithm. *Global Change Biology* **11**, 1424-1439 (2005).

77. Chapin III FS, Schulze E, Mooney HA. The ecology and economics of storage in plants. *Annual review of ecology and systematics* **21**, 423-447 (1990).

78. Iversen CM*, et al.* The unseen iceberg: plant roots in arctic tundra. *New Phytologist* **205**, 34-58 (2014).

79. Boelman NT*, et al.* Response of NDVI, biomass, and ecosystem gas exchange to long-term warming and fertilization in wet sedge tundra. *Oecologia* **135**, 414-421 (2003).

80. Street L, Shaver G, Williams M, Van Wijk M. What is the relationship between changes in canopy leaf area and changes in photosynthetic CO2 flux in arctic ecosystems? *Journal of Ecology* **95**, 139-150 (2007).

81. Goetz SJ, Prince SD. Modelling Terrestrial Carbon Exchange and Storage: Evidence and Implications of Fuctional Convergence in Light-use Efficiency. *Advances in Ecological Research* **28**, 57-92 (1999).

82. Blok D, Sass-Klaassen U, Schaepman-Strub G, Heijmans M, Sauren P, Berendse F. What are the main climate drivers for shrub growth in Northeastern Siberian tundra? *Biogeosciences* **8**, 1169-1179 (2011).

83. Andreu-Hayles L, D'Arrigo R, Anchukaitis KJ, Beck PSA, Frank D, Goetz S. Varying boreal forest response to Arctic environmental change at the Firth River, Alaska. *Environmental Research Letters* **6**, 045503 (2011).

84. Berner LT, Beck PSA, Bunn AG, Lloyd AH, Goetz SJ. High-latitude tree growth and satellite vegetation indices: Correlations and trends in Russia and Canada (1982–2008). *Journal of Geophysical Research* **116**, G01015 (2011).

85. Boelman NT, Gough L, McLaren JR, Greaves H. Does NDVI reflect variation in the structural attributes associated with increasing shrub dominance in arctic tundra? *Environmental Research Letters* **6**, 035501 (2011).

86. Epstein HE, Walker DA, Raynolds MK, Jia GJ, Kelley AM. Phytomass patterns across a temperature gradient of the North American arctic tundra. *Journal of Geophysical Research: Biogeosciences* **113**, G03S02 (2008).

87. Myers-Smith IH*, et al.* Complexity revealed in the greening of the Arctic. *Nature Climate Change* **10**, 106-117 (2020).
