# Supplementary material for: Summer warming explains widespread but not uniform greening in the Arctic tundra biome
Source: Nat Commun. 2020 Sep 22;11:4621. doi: 10.1038/s41467-020-18479-5 (PMC7509805; doi:10.1038/s41467-020-18479-5)
Supplement: Supplementary file 3 — Reporting summary [file 41467_2020_18479_MOESM3_ESM.pdf]

nature research

Corresponding author(s): Logan Berner

Last updated by author(s): 8/13/2020

Reporting Summary

Nature Research wishes to improve the reproducibility of the work that we publish. This form provides structure for consistency and transparency in reporting. For further information on Nature Research policies, see Authors & Referees and the Editorial Policy Checklist.

Statistics

For all statistical analyses, confirm that the following items are present in the figure legend, table legend, main text, or Methods section.

n/a	Confirmed	
☐	☒	The exact sample size (*n*) for each experimental group/condition, given as a discrete number and unit of measurement
☒	☐	A statement on whether measurements were taken from distinct samples or whether the same sample was measured repeatedly
☐	☒	The statistical test(s) used AND whether they are one- or two-sided<br>*Only common tests should be described solely by name; describe more complex techniques in the Methods section.*
☒	☐	A description of all covariates tested
☒	☐	A description of any assumptions or corrections, such as tests of normality and adjustment for multiple comparisons
☐	☒	A full description of the statistical parameters including central tendency (e.g. means) or other basic estimates (e.g. regression coefficient) AND variation (e.g. standard deviation) or associated estimates of uncertainty (e.g. confidence intervals)
☒	☐	For null hypothesis testing, the test statistic (e.g. *F*, *t*, *r*) with confidence intervals, effect sizes, degrees of freedom and *P* value noted<br>*Give P values as exact values whenever suitable.*
☒	☐	For Bayesian analysis, information on the choice of priors and Markov chain Monte Carlo settings
☒	☐	For hierarchical and complex designs, identification of the appropriate level for tests and full reporting of outcomes
☐	☒	Estimates of effect sizes (e.g. Cohen's *d*, Pearson's *r*), indicating how they were calculated

Our web collection on statistics for biologists contains articles on many of the points above.

Software and code

Policy information about availability of computer code

Data collection	We downloaded Landsat data from Google Earth Engine using custom code written in Python (version 3.7.3; Python Software Foundation 2018). The custom Python code will be freely available through GitHub (https://github.com/logan-berner/arctic_greening).
Data analysis	We processed, analyzed, and visualized data using the statistical software R (versions 3.5 and 3.6; R Team 2018) and a variety of add-on packages. We processed geospatial data using raster (version 3.0-12; Hijmans 2019), rgdal (version 1.4.8; Bivand et al. 2019), and maptools (version 0.9.9; Bivand and Lewin-Koh 2019). We handled data within R using data.table (version 1.12.8; Dawle and Srinivasan 2019), dplyr (version 0.8.5; Wickham and Francois 2015), and tidyr (version 1.0.2; Wickham and Henry 2020). We visualized data using lattice (version 0.20.28; Sarkar 2008), ggplot2 (version 3.3.0; Wickham 2016) and ggpubr (version 0.2.5; Kassambara 2020). We performed trend analyses using zyp (version 0.10-1.1; Bronaugh and Werner 2012) and the shrub ring analysis using dplR (version 1.7.0; Bunn 2008). We conducted the random forest modeling using randomForest (version 4.6.14; Liaw and Wiener 2002), ranger (version 0.12.1; Wright and Ziegler 2017), caret (version 6.0.86; Kuhn 2008), and pdp (version 0.7.0; Greenwell 2017). We developed custom R code carry out the study and will make all code publicly available through GitHub (https://github.com/logan-berner/arctic_greening). Lastly, we generated maps for the publication using ArcGIS (version 10.6; Redlands, CA).

For manuscripts utilizing custom algorithms or software that are central to the research but not yet described in published literature, software must be made available to editors/reviewers. We strongly encourage code deposition in a community repository (e.g. GitHub). See the Nature Research guidelines for submitting code & software for further information.

Data

Policy information about availability of data

All manuscripts must include a data availability statement. This statement should provide the following information, where applicable:

- Accession codes, unique identifiers, or web links for publicly available datasets
- A list of figures that have associated raw data
- A description of any restrictions on data availability

The data that support the findings of this study are available from the following sources: The United States Geologic Survey Landsat 5, 7, and 8 Surface Reflectance [illegible]

data are available from Google Earth Engine, https://earthengine.google.com/. The CRU TS4.01: Climatic Research Unit (CRU) Time-Series (TS) version 4.01 data are available from the Centre for Environmental Data Analysis with identifier doi:10.5285/58a8802721c94c66ae45c3baa4d814d0.The Terrestrial Air Temperature: 1900-2017 Gridded Monthly Time Series (V 5.01) data are available from the University of Delaware, http://climate.geog.udel.edu/~climate/html_pages/download.html#T2017. The Land-Ocean Temperature Index ERSSTv5 data are available from the NASA Goddard Institute for Space Studies, https://data.giss.nasa.gov/pub/gistemp/GHCNv3/gistemp1200_ERSSTv5.nc.gz. The Monthly Land + Ocean Average Temperature with Air Temperatures at Sea Ice data are available from Berkeley Earth, http://berkeleyearth.lbl.gov/auto/Global/Gridded/Land_and_Ocean_LatLong1.nc. The HadCRUT4 hybrid with UAH data are available the from University of York, https://www-users.york.ac.uk/~kdc3/papers/coverage2013/had4_short_uah_v2_0_0.nc.gz. The TerraClimate data are available from the University Corporation for Atmospheric Research, http://thredds.northwestknowledge.net:8080/thredds/catalog/TERRACLIMATE_ALL/data/catalog.html. The Arctic Circumpolar Distribution and Soil Carbon of Thermokarst Landscapes (2015) data are available from the Oak Ridge National Laboratory with identifier doi.org/10.3334/ORNLDAAC/1332. The ESA Climate Change Initiative Permafrost extent, active layer thickness, and ground temperature data are available from the Centre for Environmental Data Analysis with identifiers doi:10.5285/c7590fe40d8e44169d511c70a60ccbcc, doi:10.5285/1ee56c42cf6c4ef698693e00a63795f4, and 10.5285/c7590fe40d8e44169d511c70a60ccbcc, respectively. The ESA Climate Change Initiative Landcover data are available from the Catholic University of Louvain, http://maps.elie.ucl.ac.be/CCI/viewer/download.php. The MODIS/Terra+Aqua Burned Area Monthly L3 Global 500 m data are available from the Land Processes Distributed Active Archive Center, https://lpdaac.usgs.gov/products/mcd64a1v006/. The TanDEM-X 90m Digital Elevation Model data are available from the German Aerospace Center, https://geoservice.dlr.de/web/dataguide/tdm90/#access. The graminoid productivity data are available upon reasonable request from G.G. The shrub ring-width data are available from the (1) Polar Data Catalog with identifier, https://www.polardata.ca/pdcsearch/PDCSearchDOI.jsp?doi_id=12131, (2) the Arctic Data Center with identifiers doi:10.18739/A28Q18 and doi:10.18739/A24X0Q, and (3) the National Center for Environmental Information with identifiers https://www.ncdc.noaa.gov/paleo/study/29754, https://www.ncdc.noaa.gov/paleo/study/29752, and https://www.ncdc.noaa.gov/paleo/study/29753. Additional shrub ring-width data are available upon reasonable request from B.F. and B.G. The gross primary productivity data are available from the Arctic Observing Network, http://aon.iab.uaf.edu/data_access. Additional primary productivity data are available from Fluxnet with identifiers doi:10.18140/FLX/1440182, doi:10.18140/FLX/1440067, doi:10.18140/FLX/1440073, doi:10.18140/FLX/1440181, doi:10.18140/FLX/1440222, doi:10.18140/FLX/1440224, doi:10.18140/FLX/1440223. The Landsat datasets generated as part of this project will be publicly archived with the Oak Ridge National Laboratory Distributed Active Archive Center for Biogeochemical Dynamics.

Field-specific reporting

Please select the one below that is the best fit for your research. If you are not sure, read the appropriate sections before making your selection.

☐ Life sciences ☐ Behavioural & social sciences ☒ Ecological, evolutionary & environmental sciences

For a reference copy of the document with all sections, see nature.com/documents/nr-reporting-summary-flat.pdf

Life sciences study design

All studies must disclose on these points even when the disclosure is negative.

Sample size	*Describe how sample size was determined, detailing any statistical methods used to predetermine sample size OR if no sample-size calculation was performed, describe how sample sizes were chosen and provide a rationale for why these sample sizes are sufficient.*
Data exclusions	*Describe any data exclusions. If no data were excluded from the analyses, state so OR if data were excluded, describe the exclusions and the rationale behind them, indicating whether exclusion criteria were pre-established.*
Replication	*Describe the measures taken to verify the reproducibility of the experimental findings. If all attempts at replication were successful, confirm this OR if there are any findings that were not replicated or cannot be reproduced, note this and describe why.*
Randomization	*Describe how samples/organisms/participants were allocated into experimental groups. If allocation was not random, describe how covariates were controlled OR if this is not relevant to your study, explain why.*
Blinding	*Describe whether the investigators were blinded to group allocation during data collection and/or analysis. If blinding was not possible, describe why OR explain why blinding was not relevant to your study.*

Behavioural & social sciences study design

All studies must disclose on these points even when the disclosure is negative.

Study description	*Briefly describe the study type including whether data are quantitative, qualitative, or mixed-methods (e.g. qualitative cross-sectional, quantitative experimental, mixed-methods case study).*
Research sample	*State the research sample (e.g. Harvard university undergraduates, villagers in rural India) and provide relevant demographic information (e.g. age, sex) and indicate whether the sample is representative. Provide a rationale for the study sample chosen. For studies involving existing datasets, please describe the dataset and source.*
Sampling strategy	*Describe the sampling procedure (e.g. random, snowball, stratified, convenience). Describe the statistical methods that were used to predetermine sample size OR if no sample-size calculation was performed, describe how sample sizes were chosen and provide a rationale for why these sample sizes are sufficient. For qualitative data, please indicate whether data saturation was considered, and what criteria were used to decide that no further sampling was needed.*
Data collection	*Provide details about the data collection procedure, including the instruments or devices used to record the data (e.g. pen and paper, computer, eye tracker, video or audio equipment) whether anyone was present besides the participant(s) and the researcher, and whether the researcher was blind to experimental condition and/or the study hypothesis during data collection.*

Timing

Indicate the start and stop dates of data collection. If there is a gap between collection periods, state the dates for each sample cohort.

Data exclusions

If no data were excluded from the analyses, state so OR if data were excluded, provide the exact number of exclusions and the rationale behind them, indicating whether exclusion criteria were pre-established.

Non-participation

State how many participants dropped out/declined participation and the reason(s) given OR provide response rate OR state that no participants dropped out/declined participation.

Randomization

If participants were not allocated into experimental groups, state so OR describe how participants were allocated to groups, and if allocation was not random, describe how covariates were controlled.

Ecological, evolutionary & environmental sciences study design

All studies must disclose on these points even when the disclosure is negative.

Study description

In this study, we assessed pan-Arctic trends in tundra greenness from 1985 to 2016 using Landsat satellite observations. Furthermore, we assessed potential drivers of changes in tundra greenness as well as relationships between satellite-observed tundra greenness and field measurements of plant productivity.

Research sample

Our primary goal was to evaluate environmental changes that occurred from 1985 to 2016 in the Arctic tundra biome, specifically focusing on changes in tundra greenness and potential explanations for the observed changes. To characterize annual tundra greenness, we used time series of the Normalized Difference Vegetation Index (NDVI) derived from 30-m resolution Landsat surface reflectance data at 50,000 sampling sites in the Arctic. The NDVI has been linked to plant biomass and productivity in the Arctic and is considered an indicator of tundra greenness (see main text). The Landsat satellite provide nearly four decades of near global surface reflectance measurements at a spatial resolution that is commensurate to the grain of ecological change in the Arctic. The Landsat mission is managed jointly by NASA and USGS.

To evaluate potential explanations for observed changes in tundra greenness, we used a broad suite of existing gridded climate, permafrost, land cover, topography, and fire data sets spanning this domain. Climate data sets were interpolated from climate station measurements. Permafrost temperature and active layer thickness were modeled from satellite measurements of land surface temperature and land cover. Land cover was modeled from surface reflectance measured by multiple satellites (MERIS, AVHRR, and PROBA-V). Topography was derived from synthetic aperture radar measurements made by the TanDEM-X spacecraft. Burned area was mapped from observations made by the spaceborne Moderate Resolution Imaging Spectroradiometers (MODIS). Specific data sources are provided in the Supplementary Methods and Data Availability.

To evaluate the relationship between Landsat NDVI and plant productivity, we developed NDVI time series for field sites where there were measurements of graminoid, shrub, and ecosystem productivity. We provide extensive details about these field data in the Supplementary Methods.

Sampling strategy

We generated 50,000 terrestrial sampling sites that were randomly located within the Arctic tundra biome, as defined by Virtanen et al. (2016). We conducted a detailed analysis to ascertain how estimates of tundra greenness trends varied based on sample sizes ranging from 100 to 40,000 sample sites. This analysis revealed that trend estimates were robust with the current sample size. This analysis and the results are described in detailed in the Supplementary Methods.

Data collection

We used existing Landsat, environmental, and field data in this study. We extracted Landsat surface reflectance for each sampling site using the Python API for Google Earth Engine. We acquired the environmental data sets from publicly accessible online repositories. We acquired flux tower data from online archives operated by the Arctic Observing Network and FLUXNET. Co-author G.G. provided the graminoid productivity data set. The shrub ring width data were contributed by co-authors B.C.F., M.M.F, T.K., L.A.H., B.V.G., P.Z., R.D., and I.M.S., with further data from the Arctic Data Center.

Timing and spatial scale

The Landsat data included time series of surface reflectance collected June through August from 1985 to 2016 at 30 m spatial resolution for 50,000 sites. We chose this seasonal window to capture summer conditions and focused on these years given the availability of data. There is minimal Landsat data available in the Arctic prior to 1985. Landsat data were available through 2017 when we initiated the study, but we chose to include data through 2016 because more recent data were not uniformly available from the ensemble of climate data sets. The time series at each site was based on surface reflectance measured from all 30 m pixels within a 50 m radius (about a 3x3 pixel window). We chose to use observations from a small neighborhood to minimize effects of geolocation uncertainty. The number of Landsat satellites in orbit varied over time, with each satellite collecting measurements from a single location approximately every 14 days. The spatial coverage of Landsat observations increased substantially after 1999, especially for eastern Eurasia. We thus examined changes in tundra greenness where possible from 1985-2016 and more exhaustively across the Arctic from 2000-2016.

The climate data sets covered the pan-Arctic domain at spatial resolutions ranging from 0.2 to 2 degrees. We resampled all climate data sets to a common 50 km resolution, which was approximately the resolution of the highest-resolution climate data set. All climate data had a monthly cadence and covered at least the period from 1979-2016.

The field data sets included estimates of: (1) gross primary productivity (GPP) from flux towers; (2) graminoid aboveground net primary productivity (ANPP) from clip harvests; and (3) shrub radial growth from dendroecological techniques. Sampling characteristics varied within and among these synthetic data sets and are extensively described in the supplemental material.

Data exclusions

To generate high-quality annual time series of tundra greenness (i.e., NDVI) it was necessary to exclude Landsat data based on observation, scene, and site criteria. We excluded individual Landsat observations (i.e., a pixel at a point in time) that were affected [illegible]

by clouds, shadows, snow, or surface water as identified by the C Function of Mask (CFmask) algorithm. To minimize potential errors associated with radiometric saturation, atmospheric correction, or residual water, we also excluded observations with unrealistically high (> 1) or very low (< 0.005) surface reflectance. To minimize effects of lingering clouds and maintain geolocation accuracy, we further excluded observations from scenes with > 80% cloud cover, positional uncertainty > 30 m, or solar zenith angle > 60 degrees. These exclusion criteria were pre-established based on prior experience (e.g., Berner et al. 2018 ERL). While developing our phenology-based approach to estimate maximum summer tundra greenness, we came to the conclusion that it was necessary to exclude barren sampling sites (mean NDVI < 0.10) and sites with fewer than 30 observations or 10 years of observations. Furthermore, we focused on tundra greenness dynamics during two periods (1985-2016 and 2000-2016) and thus to help ensure the Landsat satellites adequately captured these dynamics we constrained sites used in each epochal analysis to those where the first year of Landsat observation occurred by 1986 and 2000, respectively.

Reproducibility: This is not relevant to our study because we did not perform experiments, but rather an assessment of observational trends in tundra greenness. The results of our study can be reproduced using the archived code and data sets.

Randomization: We used simple random sampling to select study sites in the Arctic tundra biome, thus enabling us to infer ecological dynamics within this domain. Furthermore, parts of the analysis involved post-stratifying these sample sites by Arctic zone to infer ecological dynamics within the High Arctic, Low Arctic, and Oro-Arctic.

Blinding: Our study did not involve blinding. We did not perform experiments with treatments, but rather assessed observational data

Did the study involve field work? ☐ Yes ☒ No

Field work, collection and transport

Field conditions: *Describe the study conditions for field work, providing relevant parameters (e.g. temperature, rainfall).*

Location: *State the location of the sampling or experiment, providing relevant parameters (e.g. latitude and longitude, elevation, water depth).*

Access and import/export: *Describe the efforts you have made to access habitats and to collect and import/export your samples in a responsible manner and in compliance with local, national and international laws, noting any permits that were obtained (give the name of the issuing authority, the date of issue, and any identifying information).*

Disturbance: *Describe any disturbance caused by the study and how it was minimized.*

Reporting for specific materials, systems and methods

We require information from authors about some types of materials, experimental systems and methods used in many studies. Here, indicate whether each material, system or method listed is relevant to your study. If you are not sure if a list item applies to your research, read the appropriate section before selecting a response.

Materials & experimental systems

n/a	Involved in the study	
☒	☐	Antibodies
☒	☐	Eukaryotic cell lines
☒	☐	Palaeontology
☒	☐	Animals and other organisms
☒	☐	Human research participants
☒	☐	Clinical data

Methods

n/a	Involved in the study	
☒	☐	ChIP-seq
☒	☐	Flow cytometry
☒	☐	MRI-based neuroimaging

Antibodies

Antibodies used: *Describe all antibodies used in the study; as applicable, provide supplier name, catalog number, clone name, and lot number.*

Validation: *Describe the validation of each primary antibody for the species and application, noting any validation statements on the manufacturer's website, relevant citations, antibody profiles in online databases, or data provided in the manuscript.*

Eukaryotic cell lines

Policy information about cell lines

Cell line source(s): *State the source of each cell line used.*

Authentication: *Describe the authentication procedures for each cell line used OR declare that none of the cell lines used were authenticated.*

Mycoplasma contamination: *Confirm that all cell lines tested negative for mycoplasma contamination OR describe the results of the testing for mycoplasma contamination OR declare that the cell lines were not tested for mycoplasma contamination.*

Commonly misidentified lines (See ICLAC register) | *Name any commonly misidentified cell lines used in the study and provide a rationale for their use.*

Palaeontology

Specimen provenance | *Provide provenance information for specimens and describe permits that were obtained for the work (including the name of the issuing authority, the date of issue, and any identifying information).*

Specimen deposition | *Indicate where the specimens have been deposited to permit free access by other researchers.*

Dating methods | *If new dates are provided, describe how they were obtained (e.g. collection, storage, sample pretreatment and measurement), where they were obtained (i.e. lab name), the calibration program and the protocol for quality assurance OR state that no new dates are provided.*

☐ Tick this box to confirm that the raw and calibrated dates are available in the paper or in Supplementary Information.

Animals and other organisms

Policy information about studies involving animals; ARRIVE guidelines recommended for reporting animal research

Laboratory animals | *For laboratory animals, report species, strain, sex and age OR state that the study did not involve laboratory animals.*

Wild animals | *Provide details on animals observed in or captured in the field; report species, sex and age where possible. Describe how animals were caught and transported and what happened to captive animals after the study (if killed, explain why and describe method; if released, say where and when) OR state that the study did not involve wild animals.*

Field-collected samples | *For laboratory work with field-collected samples, describe all relevant parameters such as housing, maintenance, temperature, photoperiod and end-of-experiment protocol OR state that the study did not involve samples collected from the field.*

Ethics oversight | *Identify the organization(s) that approved or provided guidance on the study protocol, OR state that no ethical approval or guidance was required and explain why not.*

Note that full information on the approval of the study protocol must also be provided in the manuscript.

Human research participants

Policy information about studies involving human research participants

Population characteristics | *Describe the covariate-relevant population characteristics of the human research participants (e.g. age, gender, genotypic information, past and current diagnosis and treatment categories). If you filled out the behavioural & social sciences study design questions and have nothing to add here, write "See above."*

Recruitment | *Describe how participants were recruited. Outline any potential self-selection bias or other biases that may be present and how these are likely to impact results.*

Ethics oversight | *Identify the organization(s) that approved the study protocol.*

Note that full information on the approval of the study protocol must also be provided in the manuscript.

Clinical data

Policy information about clinical studies

All manuscripts should comply with the ICMJE guidelines for publication of clinical research and a completed CONSORT checklist must be included with all submissions.

Clinical trial registration | *Provide the trial registration number from ClinicalTrials.gov or an equivalent agency.*

Study protocol | *Note where the full trial protocol can be accessed OR if not available, explain why.*

Data collection | *Describe the settings and locales of data collection, noting the time periods of recruitment and data collection.*

Outcomes | *Describe how you pre-defined primary and secondary outcome measures and how you assessed these measures.*

ChIP-seq

Data deposition

☐ Confirm that both raw and final processed data have been deposited in a public database such as GEO.

☐ Confirm that you have deposited or provided access to graph files (e.g. BED files) for the called peaks.

Data access links
May remain private before publication. | *For "Initial submission" or "Revised version" documents, provide reviewer access links. For your "Final submission" document, provide a link to the deposited data.*

Files in database submission | *Provide a list of all files available in the database submission.*

Genome browser session (e.g. UCSC) | *Provide a link to an anonymized genome browser session for "Initial submission" and "Revised version" documents only, to enable peer review. Write "no longer applicable" for "Final submission" documents.*

Methodology

Replicates | *Describe the experimental replicates, specifying number, type and replicate agreement.*

Sequencing depth | *Describe the sequencing depth for each experiment, providing the total number of reads, uniquely mapped reads, length of reads and whether they were paired- or single-end.*

Antibodies | *Describe the antibodies used for the ChIP-seq experiments; as applicable, provide supplier name, catalog number, clone name, and lot number.*

Peak calling parameters | *Specify the command line program and parameters used for read mapping and peak calling, including the ChIP, control and index files used.*

Data quality | *Describe the methods used to ensure data quality in full detail, including how many peaks are at FDR 5% and above 5-fold enrichment.*

Software | *Describe the software used to collect and analyze the ChIP-seq data. For custom code that has been deposited into a community repository, provide accession details.*

Flow Cytometry

Plots

Confirm that:

- [ ] The axis labels state the marker and fluorochrome used (e.g. CD4-FITC).
- [ ] The axis scales are clearly visible. Include numbers along axes only for bottom left plot of group (a 'group' is an analysis of identical markers).
- [ ] All plots are contour plots with outliers or pseudocolor plots.
- [ ] A numerical value for number of cells or percentage (with statistics) is provided.

Methodology

Sample preparation | *Describe the sample preparation, detailing the biological source of the cells and any tissue processing steps used.*

Instrument | *Identify the instrument used for data collection, specifying make and model number.*

Software | *Describe the software used to collect and analyze the flow cytometry data. For custom code that has been deposited into a community repository, provide accession details.*

Cell population abundance | *Describe the abundance of the relevant cell populations within post-sort fractions, providing details on the purity of the samples and how it was determined.*

Gating strategy | *Describe the gating strategy used for all relevant experiments, specifying the preliminary FSC/SSC gates of the starting cell population, indicating where boundaries between "positive" and "negative" staining cell populations are defined.*

- [ ] Tick this box to confirm that a figure exemplifying the gating strategy is provided in the Supplementary Information.

Magnetic resonance imaging

Experimental design

Design type | *Indicate task or resting state; event-related or block design.*

Design specifications | *Specify the number of blocks, trials or experimental units per session and/or subject, and specify the length of each trial or block (if trials are blocked) and interval between trials.*

Behavioral performance measures | *State number and/or type of variables recorded (e.g. correct button press, response time) and what statistics were used to establish that the subjects were performing the task as expected (e.g. mean, range, and/or standard deviation across subjects).*

Acquisition

Imaging type(s)

Specify: functional, structural, diffusion, perfusion.

Field strength

Specify in Tesla

Sequence & imaging parameters

Specify the pulse sequence type (gradient echo, spin echo, etc.), imaging type (EPI, spiral, etc.), field of view, matrix size, slice thickness, orientation and TE/TR/flip angle.

Area of acquisition

State whether a whole brain scan was used OR define the area of acquisition, describing how the region was determined.

Diffusion MRI ☐ Used ☐ Not used

Preprocessing

Preprocessing software

Provide detail on software version and revision number and on specific parameters (model/functions, brain extraction, segmentation, smoothing kernel size, etc.).

Normalization

If data were normalized/standardized, describe the approach(es): specify linear or non-linear and define image types used for transformation OR indicate that data were not normalized and explain rationale for lack of normalization.

Normalization template

Describe the template used for normalization/transformation, specifying subject space or group standardized space (e.g. original Talairach, MNI305, ICBM152) OR indicate that the data were not normalized.

Noise and artifact removal

Describe your procedure(s) for artifact and structured noise removal, specifying motion parameters, tissue signals and physiological signals (heart rate, respiration).

Volume censoring

Define your software and/or method and criteria for volume censoring, and state the extent of such censoring.

Statistical modeling & inference

Model type and settings

Specify type (mass univariate, multivariate, RSA, predictive, etc.) and describe essential details of the model at the first and second levels (e.g. fixed, random or mixed effects; drift or auto-correlation).

Effect(s) tested

Define precise effect in terms of the task or stimulus conditions instead of psychological concepts and indicate whether ANOVA or factorial designs were used.

Specify type of analysis: ☐ Whole brain ☐ ROI-based ☐ Both

Statistic type for inference
(See Eklund et al. 2016)

Specify voxel-wise or cluster-wise and report all relevant parameters for cluster-wise methods.

Correction

Describe the type of correction and how it is obtained for multiple comparisons (e.g. FWE, FDR, permutation or Monte Carlo).

Models & analysis

n/a	Involved in the study
☐	☐ Functional and/or effective connectivity
☐	☐ Graph analysis
☐	☐ Multivariate modeling or predictive analysis

Functional and/or effective connectivity

Report the measures of dependence used and the model details (e.g. Pearson correlation, partial correlation, mutual information).

Graph analysis

Report the dependent variable and connectivity measure, specifying weighted graph or binarized graph, subject- or group-level, and the global and/or node summaries used (e.g. clustering coefficient, efficiency, etc.).

Multivariate modeling and predictive analysis

Specify independent variables, features extraction and dimension reduction, model, training and evaluation metrics.
